# A single amino acid substitution in the movement protein enables the mechanical transmission of a geminivirus

**DOI:** 10.1111/mpp.12917

**Published:** 2020-02-20

**Authors:** Chia‐Hwa Lee, You‐Xiu Zheng, Chin‐Hsiang Chan, Hsin‐Mei Ku, Chung‐Jan Chang, Fuh‐Jyh Jan

**Affiliations:** ^1^ Ph.D. Program in Microbial Genomics National Chung Hsing University and Academia Sinica Taichung Taipei Taiwan; ^2^ Department of Plant Pathology National Chung Hsing University Taichung Taiwan; ^3^ Department of Agronomy National Chung Hsing University Taichung Taiwan; ^4^ Department of Plant Pathology University of Georgia Griffin USA; ^5^ Advanced Plant Biotechnology Center National Chung Hsing University Taichung Taiwan

**Keywords:** begomovirus, mechanical transmissibility, movement protein, ToLCNDV oriental melon isolate, tomato leaf curl New Delhi virus

## Abstract

Begomoviruses of the *Geminiviridae* are usually transmitted by whiteflies and rarely by mechanical inoculation. We used tomato leaf curl New Delhi virus (ToLCNDV), a bipartite begomovirus, to address this issue. Most ToLCNDV isolates are not mechanically transmissible to their natural hosts. The ToLCNDV‐OM isolate, originally identified from a diseased oriental melon plant, is mechanically transmissible, while the ToLCNDV‐CB isolate, from a diseased cucumber plant, is not. Genetic swapping and pathological tests were performed to identify the molecular determinants involved in mechanical transmission. Various viral infectious clones were constructed and successfully introduced into *Nicotiana benthamiana*, oriental melon, and cucumber plants by *Agrobacterium*‐mediated inoculation. Mechanical transmissibility was assessed via direct rub inoculation with sap prepared from infected *N. benthamiana*. The presence or absence of viral DNA in plants was validated by PCR, Southern blotting, and in situ hybridization. The results reveal that mechanical transmissibility is associated with the movement protein (MP) of viral DNA‐B in ToLCNDV‐OM. However, the nuclear shuttle protein of DNA‐B plays no role in mechanical transmission. Analyses of infectious clones carrying a single amino acid substitution reveal that the glutamate at amino acid position 19 of MP in ToLCNDV‐OM is critical for mechanical transmissibility. The substitution of glutamate with glycine at this position in the MP of ToLCNDV‐OM abolishes mechanical transmissibility. In contrast, the substitution of glycine with glutamate at the 19th amino acid position in the MP of ToLCNDV‐CB enables mechanical transmission. This is the first time that a specific geminiviral movement protein has been identified as a determinant of mechanical transmissibility.

## Graphical abstract

Begomoviruses are usually transmitted by whiteflies and rarely by mechanical inoculation. The movement protein of tomato leaf curl New Delhi virus is identified as a determinant of mechanical transmissibility.

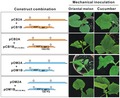

## 1 INTRODUCTION

Begomoviruses cause epidemics in many economically important crops worldwide, including cassava, cucurbits, eggplant, legumes, okra, pepper, potato, and tomato (Green *et al.*, 2003; Varma and Malathi, 2003; Shih *et al.*, 2007). The infection of cassava by African cassava mosaic virus (ACMV) causes annual economic losses of $1.9–2.7 billion in Africa (Legg and Fauquet, 2004). The infection of sugar beet by bean golden mosaic virus (BGMV) and tomato by tomato yellow leaf curl virus (TYLCV) also leads to a severe reduction in crop yields (Varma and Malathi, 2003).

One of the most important factors contributing to viral epidemics is the mode of transmission. Comprehensive knowledge of viral transmission mechanisms and their potential modifications during viral evolution may lead to the development of more effective preventive measures. Begomoviruses are usually transmitted by whiteflies (*Bemisia tabaci*). Similar to many phloem‐limited viruses, including other geminiviruses and closteroviruses, begomoviruses are rarely mechanically transmissible by rub or sap inoculation (Garnsey *et al.*, 1977; Wege and Pohl, 2007). Thus far, only approximately 20 out of 409 known begomovirus species have been reported to be mechanically transmissible to their natural hosts (Bock and Guthrie, 1978; Morales *et al.*, 1990; Gilbertson *et al.*, 1991; Garrido‐Ramirez *et al.*, 2000; Chatchawankanphanich and Maxwell, 2002; Usharani *et al.*, 2004; Ajlan *et al.*, 2007; Tsai *et al.*, 2011).

The mechanisms underlying mechanical or nonmechanical transmissibility among related viruses of the same genus are of significant interest from the point of view of evolution and epidemics. The identification of the viral determinants associated with mechanical transmissibility is a critical step towards mechanistic studies. Begomoviruses have a single‐stranded circular monopartite (DNA‐A like) or bipartite (DNA‐A and DNA‐B) genome (Fauquet *et al.*, 2008). The DNA‐A genome encodes proteins required for virus replication and encapsidation, whereas DNA‐B encodes proteins responsible for viral movement between subcellular and intercellular compartments as well as pathogenicity (Krenz *et al.*, 2012). Although the DNA‐B genomes of some begomoviruses have been implicated in mechanical transmission (Levy and Czosenk, 2003; Wege and Pohl, 2007), the specific viral proteins involved in this aspect remain largely unknown.

Tomato leaf curl New Delhi virus (ToLCNDV) is a begomovirus with a bipartite genome. This virus could cause severe damage to many economically important crops in the families of Solanaceae and Cucurbitaceae, including tomato, pepper, potato, cucumber, melon, sponge gourd, and pumpkin (Padidam *et al.*, 1995; Samretwanich *et al.*, 2000; Usharani *et al.*, 2004; Hussain *et al.*, 2005; Ito *et al*., 2008; Khan *et al.*, 2012; Lopez *et al.*, 2015). ToLCNDV DNA‐A has two open reading frames (ORFs; *AV1* and *AV2*) on the viral‐sense strand and four ORFs (*AC1* to *AC4*) on the antisense strand. *AV1* encodes a coat protein (CP) and *AV2* encodes a pre‐coat protein. *AC1*, *AC2*, and *AC3* encode a replication‐associated protein (Rep), a transcriptional activator protein (TrAP), and a replication enhancer (REn), respectively. *AC4* encodes a protein required for symptom development (Fondong, 2013). DNA‐B has two ORFs: *BV1* of the viral‐sense strand encodes a nuclear shuttle protein (NSP) and *BC1* of the antisense strand encodes a cell‐to‐cell movement protein (MP) (Jeske, 2009).

ToLCNDV is transmitted by whiteflies in nature. However, greenhouse tests have revealed that some ToLCNDV isolates can be mechanically transmitted by rub inoculation while others cannot. The ToLCNDV‐OM isolate, originally identified from a diseased oriental melon plant, can infect oriental melon, pickling melon, bottle gourd, cucumber, zucchini, and luffa via mechanical sap inoculation (Chang *et al.*, 2010). The ToLCNDV‐potato isolate can also be transmitted to host plants via mechanical inoculation (Usharani *et al.*, 2004). In contrast, the ToLCNDV‐severe and ToLCNDV‐cucumber isolates, which share a very similar genome organization and size with oriental melon and potato isolates, cannot be mechanically transmitted to their hosts (Padidam *et al.*, 1995; Samretwanich *et al.*, 2000). The divergent modes of mechanical transmissibility among ToLCNDV isolates may be due to the high genomic recombination and mutation rates of geminiviruses, which could lead to the emergence of a new isolate with different transmission abilities or host ranges (Chatchawankanphanich and Maxwell, 2002). New viral variants are often more pathogenic or can exhibit a wider host range than previously existing variants (Arguello‐Astorga *et al.*, 2007; Duffy and Holmes, 2008). For example, tomato yellow leaf curl Thailand virus (TYLCTHV) is mechanically transmissible and has spread widely to become a predominant strain in Taiwan (Tsai *et al.*, 2011). In contrast, an endogenous tomato leaf curl Taiwan virus (ToLCTWV), which is not mechanically transmissible, has become less common in the region.

The mechanisms of the mechanical transmission of begomoviruses have not received much attention, because many of them are transmitted by whiteflies. The new ToLCNDV‐CB isolate, recently identified from a cucumber plant with symptoms, shares high genome sequence similarity with the ToLCNDV‐OM isolate. Unlike ToLCNDV‐OM, the ToLCNDV‐CB isolate cannot be transmitted to host plants by sap inoculation. In this study, genetic and pathological approaches were employed to identify the molecular determinants responsible for the mechanical transmission of ToLCNDV‐OM. Gene swapping and point mutations revealed that the 19th amino acid residue at the N‐terminus of the MP of DNA‐B plays a critical role in mechanical transmission. The results also showed that the NSP‐coding region plays no role in mechanical transmission.

## 2 RESULTS

### 2.1 ToLCNDV isolates share high sequence similarity

The ToLCNDV‐OM isolate, originally obtained from a diseased oriental melon, could be mechanically transmitted to its host plants. In contrast, the closely related ToLCNDV‐CB isolate, originally collected from cucumber with symptoms, failed to infect *Nicotiana benthamiana*, oriental melon, or cucumber plants by mechanical sap inoculation. Similar to ToLCNDV‐OM, the ToLCNDV‐CB isolate was found to have a bipartite genome consisting of DNA‐A (2,738 nt) and DNA‐B (2,695 nt). Pairwise sequence comparisons of the ToLCNDV‐CB genome with the ToLCNDV‐OM, ToLCNDV‐severe (nonmechanically transmissible), and ToLCNDV‐potato (mechanically transmissible) genome sequences available in GenBank were conducted to determine their genetic relationships. The whole‐DNA‐A‐genome sequences of the ToLCNDV isolates shared from 92.8% to 96.5% nucleotide identity, and those of DNA‐B shared from 84.4% to 92.3% nucleotide identity. The amino acid sequences encoded by DNA‐A or DNA‐B were highly similar, with the lowest similarity found among AC4 (88.1%–91.5%) of DNA‐A and BC1 (88.7%–97.2%) of DNA‐B (Table 1). ToLCNDV‐OM and ToLCNDV‐CB showed 96.5% identity of their DNA‐A sequences and 92.3% of their DNA‐B genomes. In addition to their high sequence similarity, the two isolates were found to induce similar symptoms and present very similar host ranges (Figure S1).

**Table 1:** Sequence similarity of full‐length DNA‐A and DNA‐B and individual genes of the tomato leaf curl New Delhi virus (ToLCNDV)‐OM with those of other ToLCNDV isolates

Isolate	Sequence identity and similarity (%)									
	DNA‐A	AV1	AV2	AC1	AC2	AC3	AC4	DNA‐B	BV1	BC1
CB	96.5^a^	99.0 (99.6)^b^	97.6 (99.1)	95.1 (96.1)	98.3 (96.3)	97.3 (97.8)	97.2 (91.5)	92.3	94.3 (95.2)	95.4 (97.2)
Severe	93.4	95.2 (97.7)	94.4 (95.6)	93.2 (93.9)	95.6 (92.6)	95.9 (97.1)	94.9 (88.1)	85.1	87.5 (89.6)	89.7 (88.7)
Potato	92.8	94.0 (95.7)	94.4 (93.8)	93.2 (94.8)	95.3 (90.4)	93.4 (91.2)	95.5 (89.8)	84.4	87.7 (90.0)	90.2 (94.0)

Note

The sequences used in this study can be retrieved from the GenBank database: ToLCNDV‐OM isolate (DNA‐A, accession number GU180095; DNA‐B, accession number MK883714); ToLCNDV‐CB isolate (DNA‐A, accession number MK883715; DNA‐B, accession number MK883716); ToLCNDV‐severe isolate (DNA‐A, accession number U15015; DNA‐B, accession number U15017), and ToLCNDV‐potato isolate (DNA‐A, accession number AY286316; DNA‐B, accession number AY158080).

a The identity of nucleotide sequences.

b The similarity of amino acids.

### 2.2 The DNA‐B genome of ToLCNDV‐OM is required for mechanical transmissibility

**Figure 1:**
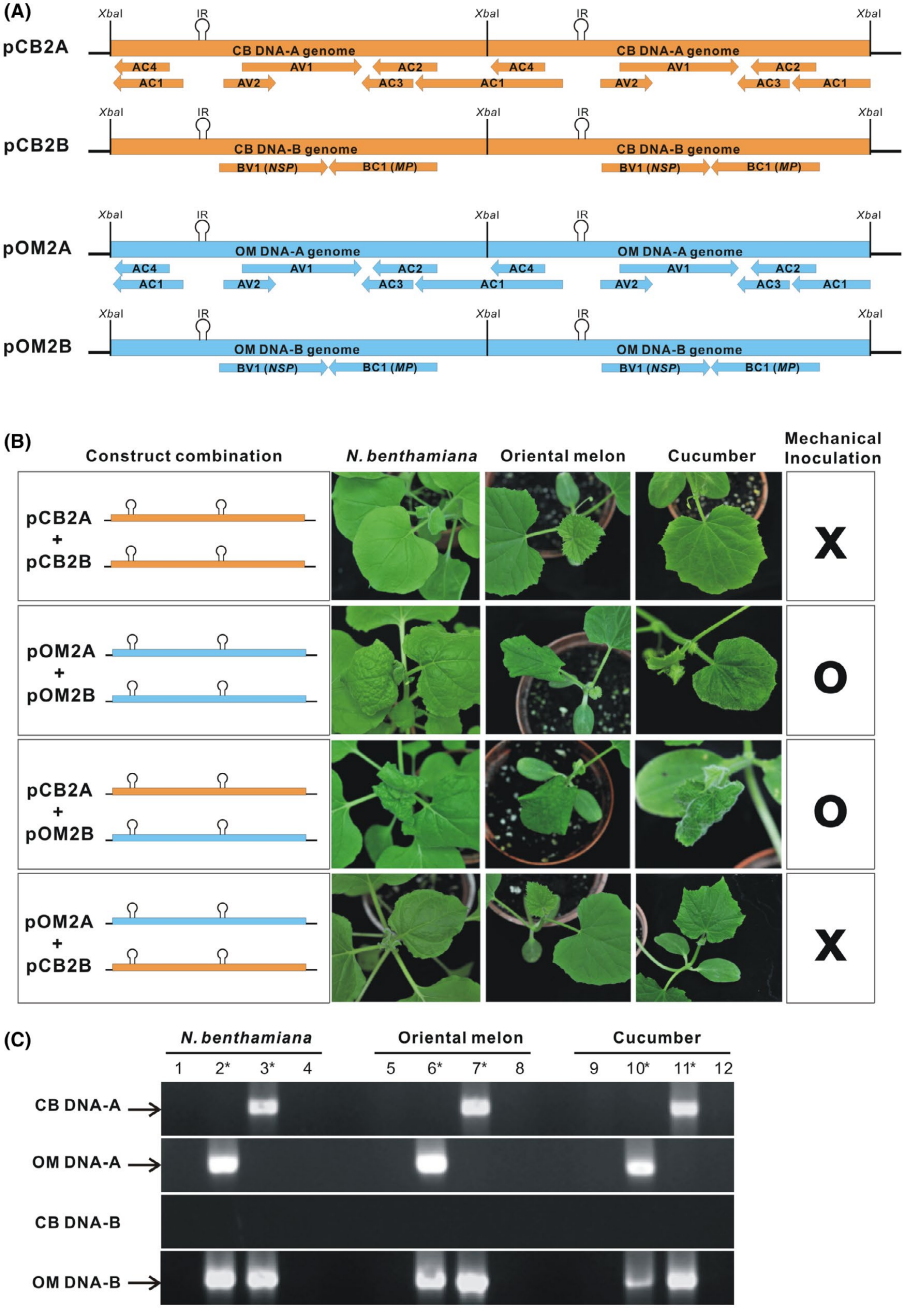
The DNA‐B genome of the tomato leaf curl New Delhi virus (ToLCNDV)‐OM isolate is required for mechanical transmissibility. (a) Physical maps of DNA infectious clones (pCB2A, pCB2B, pOM2A, and pOM2B) carrying the duplicated DNA‐A or DNA‐B genome of the ToLCNDV‐CB or ToLCNDV‐OM isolate. Genes encoded by DNA‐A or DNA‐B and the location of the intergenic region (IR) sequence are also indicated. The arrow indicates the direction of the transcription of each gene. (b) Images of *Nicotiana benthamiana* (10 days post‐inoculation, dpi), oriental melon, and cucumber plants (12 dpi) after mechanical inoculation with the wild‐type virus (pCB2A + pCB2B and pOM2A + pOM2B) or pseudorecombinant viruses (pCB2A + pOM2B and pOM2A + pCB2B) developing viral symptoms (O) or exhibiting no symptoms (X). The viral inoculum used for mechanical sap inoculation was prepared from diseased *N. benthamiana* after agroinoculation with an appropriate combination of the clones. (c) PCR detection of virus accumulation in the leaves after mechanical inoculation with pCB2A + pCB2B (lanes 1, 5, and 9), pOM2A + pOM2B (lanes 2, 6, and 10), pCB2A + pOM2B (lanes 3, 7, and 11), and pOM2A + pCB2B (lanes 4, 8, and 12). The specific DNA fragments amplified by PCR are indicated on the left. The pseudoinfectious clones leading to successful mechanical sap inoculation are indicated by asterisks

Because ToLCNDV‐CB was unable to infect oriental melon and *N. benthamiana* by mechanical sap inoculation, infectious clones of ToLCNDV‐CB DNA‐A and DNA‐B amplified via the rolling circle amplification (RCA) method were independently constructed in the binary vector pCAMBIA1304 (Figure 1) for further tests. Each clone contained two copies of the respective DNA sequences, and they were thus designated pCB2A (DNA‐A) and pCB2B (DNA‐B). The pCB2A and pCB2B infectious clones were successfully introduced into *N. benthamiana*, oriental melon, and cucumber by agroinoculation, resulting in visible symptoms (Table 2). Viral symptoms including curling, puckering, and mosaic appeared on *N. benthamiana* leaves 5–7 days post‐inoculation (dpi) and on oriental melon and cucumber leaves 7–10 dpi. However, sap prepared from diseased *N. benthamiana* leaves inoculated with pCB2A and pCB2B failed to induce symptoms in any of the test plants after rub inoculation. The ToLCNDV‐OM infectious clones pOM2A and pOM2B, constructed in our previous study (Chang *et al.*, 2010), were also introduced into *N. benthamiana*, oriental melon, and cucumber by agroinoculation and induced symptoms at similar rates and with similar magnitudes to those induced by the ToLCNDV‐CB clones. Sap prepared from the ToLCNDV‐O‐infected *N. benthamiana* leaves also resulted in symptoms on the test plants following rub inoculation at incidence rates ranging from 40% to 85% (Table 2). Viral DNA‐A and DNA‐B in the plant leaves with symptoms were monitored by PCR amplification using genome‐specific primers, resulting in products with similar intensities.

**Table 2:** Summary of infection results for the tomato leaf curl New Delhi virus (ToLCNDV) isolates, pseudorecombinants, and mutants

DNA‐A^a^	DNA‐B	Agroinoculation			Mechanical inoculation^c^		
		*Nicotiana benthamiana*	Oriental melon	Cucumber	*Nicotiana benthamiana*	Oriental melon	Cucumber
pCB2A	pCB2B	10/10	11/12 (91.7%)	11/12 (91.7%)	0/26	0/25	0/24
pOM2A	pOM2B	10/10	11/12 (91.7%)	10/12 (83.3%)	22/26 (84.6%)	10/25 (40.0%)	11/25 (44.0%)
pCB2A	pOM2B	10/10	11/12 (91.7%)	12/12	19/26 (73.1%)	11/26 (42.3%)	10/27 (37.0%)
pOM2A	pCB2B	9/10	10/12 (83.3%)	11/12 (91.7%)	0/26	0/25	0/25
pOM2A	pOM1B::*NSP*_ins39_	5/5	2/5 (40.0%)	‐	6/6	5/8 (62.5%)	‐
pCB2A	pCB1B	10/10	15/17 (88.2%)	10/11 (90.9%)	0/26	0/30	0/28
pOM2A	pOM1B	10/10	11/13 (84.6%)	13/13	21/26 (80.8%)	17/29 (58.6%)	14/27 (51.9%)
pCB2A	pCB1B::OM*_NSP_*	9/10 (90.0%)^b^	11/13 (84.6%)	10/12 (83.3%)	0/26	0/29	0/27
pOM2A	pOM1B::CB*_NSP_*	10/10	12/13 (92.3%)	12/13 (92.3%)	22/26 (84.6%)	16/29 (55.2%)	12/27 (44.4%)
pCB2A	pCB1B::OM*_MP_*	12/12	17/17	10/11 (90.9%)	23/26 (88.5%)	14/30 (46.7%)	12/30 (40.0%)
pOM2A	pOM1B::CB*_MP_*	10/10	12/13 (92.3%)	13/13	0/26	0/29	0/27
pCB2A	pCB1B::OM_5′_ *_MP_*	8/8	–d	–	7/8 (87.5%)	4/8 (50.0%)	3/8 (37.5%)
pCB2A	pCB1B::OM_3′_ *_MP_*	8/8	–	–	0/8	0/8	0/8
pOM2A	pOM1B::CB_5′_ *_MP_*	8/8	–	–	0/8	0/8	0/8
pOM2A	pOM1B::CB_3′_ *_MP_*	8/8	–	–	6/8 (75.0%)	3/8 (37.5%)	4/8 (50.0%)
pCB2A	pCB1B*_MP_*_(3I→T)_	8/8	–	–	0/8	0/9	0/5
pCB2A	pCB1B*_MP_*_(6D→E)_	8/8	–	–	0/8	0/12	0/12
pCB2A	pCB1B*_MP_*_(8V→M)_	8/8	–	–	0/8	0/9	0/5
pCB2A	pCB1B*_MP_*_(19G→E)_	8/8	–	–	7/8 (87.5%)	6/9 (66.7%)	1/5 (20.0%)
pOM2A	pOM1B*_MP_*_(3T→I)_	8/8	–	–	8/8	7/9 (77.8%)	1/5 (20.0%)
pOM2A	pOM1B*_MP_*_(6E→D)_	8/8	–	–	7/8 (87.5%)	3/12 (25.0%)	9/12 (75.0%)
pOM2A	pOM1B*_MP_*_(8M→V)_	8/8	–	–	7/8 (87.5%)	6/9 (66.7%)	1/5 (20.0%)
pOM2A	pOM1B*_MP_*_(19E→G)_	8/8	–	–	0/8	0/9	0/5

Note

Abbreviations for amino acids: G, glycine; E, glutamate; I, isoleucine; T, threonine; D, aspartic acid; V, valine; M, methionine.

a Detailed construction maps of the infectious DNA‐A and ‐B are illustrated in Figures 1a, 3a, 4a and 5.

b Disease incidence (%) is indicated by number of infected plants/total number of plants inoculated.

c The sap was prepared from the agroinoculated *N. benthamiana* and used as inoculum for mechanical inoculation.

d The symbol “–“ indicates that infectivity was not determined.

To determine whether DNA‐A or DNA‐B plays a role in mechanical transmission, a combination of DNA‐A and DNA‐B from ToLCNDV‐CB and ToLCNDV‐OM was tested for infectivity via mechanical sap inoculation. When the test plants were infected with the pCB2A + pOM2B or pOM2A + pCB2B pseudorecombinants by agroinoculation, most developed symptoms at rates ranging from 83.3% to 100% (Table 2). When sap extracted from diseased *N. benthamiana* leaves infected with pCB2A + pOM2B was rub inoculated onto the test plants, many of them also developed symptoms at rates (37% to 73%) comparable to those in plants inoculated with pOM2A + pOM2B. In contrast, sap extracted from *N. benthamiana* infected with pOM2A + pCB2B failed to induce symptoms on the test plants after rub inoculation (Figure 1b), indicating a role of DNA‐B from the ToLCNDV‐OM isolate in mechanical transmissibility. The presence of both DNA‐A and DNA‐B in sap‐inoculated plants was further confirmed by PCR using genome‐specific primers (Figure 1c).

### 2.3 The *NSP* gene of ToLCNDV‐OM plays no role in mechanical transmissibility

**Figure 2:**
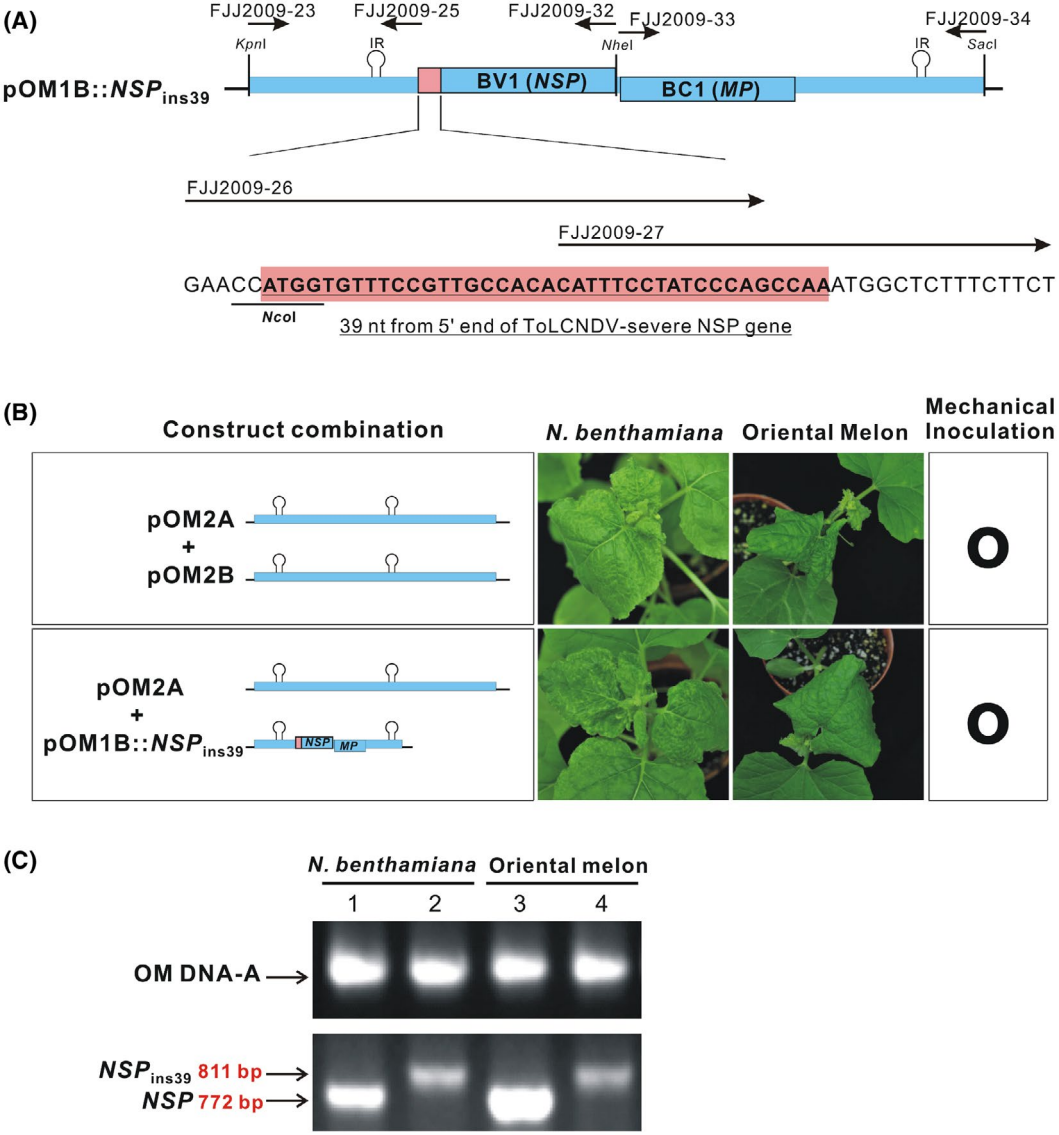
The addition of 39 nucleotides of the *NSP* gene of the tomato leaf curl New Delhi virus (ToLCNDV)‐OM has no effect on mechanical transmissibility. (a) Physical map of the pOM1B::*NSP*_ins39_ infectious clone constructed in the ToLCNDV‐OM DNA‐B genome (pOM1B) by adding an extra 39‐nt fragment (indicated by a pink box) from the *NSP* gene of the ToLCNDV‐severe isolate by PCR using two overlapping primers (FJJ2009‐26 and FJJ2009‐27). A recognition site for the *Nco*I endonuclease was incorporated into FJJ2009‐26 to facilitate cloning. Other primers used for cloning and the location of the intergenic region (IR) are also indicated. (b) Symptoms of *Nicotiana benthamiana* and oriental melon after sap inoculation with wild‐type ToLCNDV‐OM (pOM2A + pOM2B) and the *NSP* mutant (pOM2A + pOM1B::*NSP*_ins39_) at 10 days post‐inoculation. The viral inoculum used for mechanical sap inoculation was prepared from *N. benthamiana* with symptoms after agroinoculation with an appropriate combination of the clones. (c) Detection of viral DNA using PCR. Specific primers (Table S1) were used to amplify DNA‐A (upper panel) and DNA‐B (lower panel). An 772‐bp fragment representing the DNA‐B genome of the wild‐type virus was amplified from plants mechanically inoculated with pOM2A + pOM2B (lanes 1 and 3). A 811‐bp fragment representing the DNA‐B genome of pOM1B::*NSP*_ins39_ was amplified from plants inoculated with pOM2A + pOM1B::*NSP*_ins39_ (lanes 2 and 4)

Sequence alignment has shown that the *NSP* genes of ToLCNDV‐OM and ToLCNDV‐potato (both mechanically transmissible isolates) are 39 nt shorter than that of the ToLCNDV‐severe isolate, which is not mechanically transmissible (Chang *et al.*, 2010). To determine whether the addition of 39 nt at the 5′ end of the *NSP* gene of ToLCNDV‐severe plays a negative role in mechanical transmission, the pOM1B::*NSP*_ins39_ clone (Figure 2a), containing an extra 39‐nt fragment from the *NSP* gene of the ToLCNDV‐severe isolate, was constructed in the DNA‐B genome of ToLCNDV‐OM. The agroinoculation of test plants with pOM2A + pOM1B::*NSP*_ins39_ resulted in a high level of infectivity in *N. benthamiana* and oriental melon (Table 2). Sap prepared from *N. benthamiana* leaves with symptoms infected with pOM2A + pOM1B::*NSP*_ins39_ produced visible symptoms on two plant species following rub inoculation (Figure 2b), indicating that the addition of the 39 nt had no negative effect on mechanical transmissibility. The presence of ToLCNDV‐OM DNA‐A and DNA‐B in the mechanically infected plants was also confirmed by PCR using DNA‐specific primers. The DNA‐B primers amplified the expected 772‐bp fragment from pOM2B and 811‐bp fragment from pOM1B::*NSP*_ins39_ (Figure 2c).

**Figure 3:**
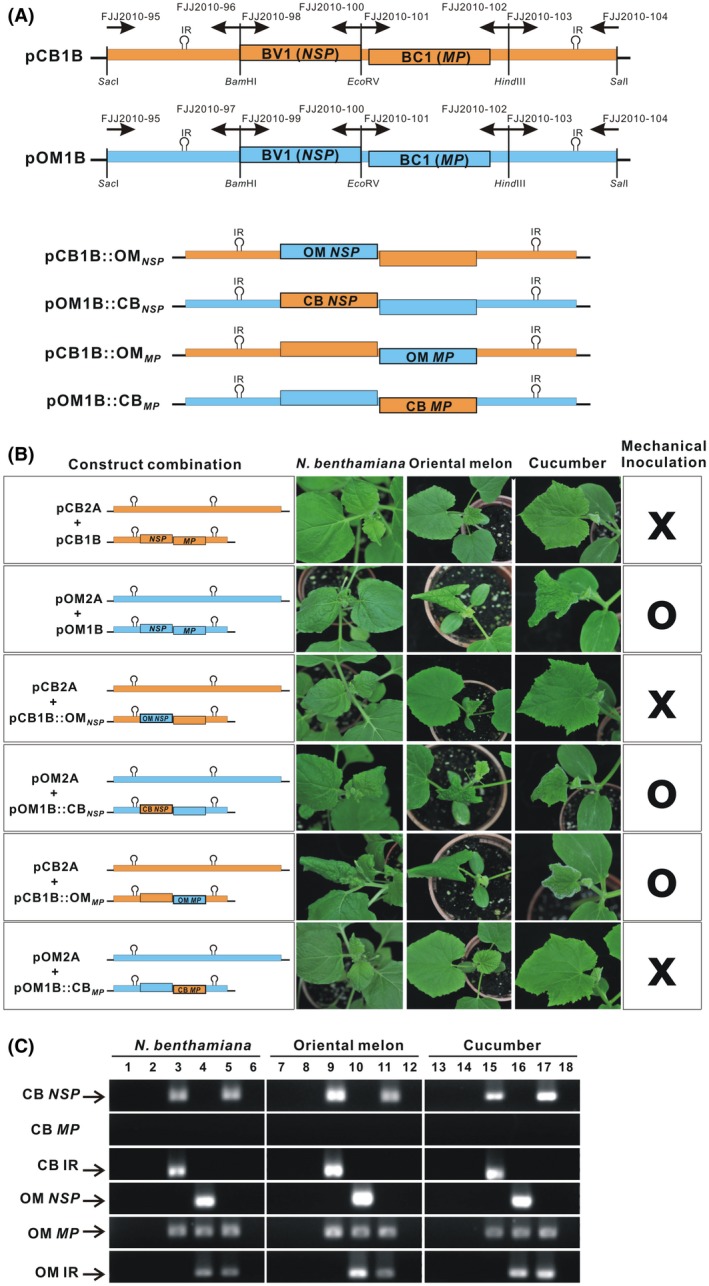
The tomato leaf curl New Delhi virus (ToLCNDV)‐OM movement protein (MP), but not nuclear shuttle protein (NSP), is required for mechanical transmission. (a) Schematic illustration of the pCB1B and pOM1B infectious clones carrying a single copy of the DNA‐B genome of the CB or OM isolate, respectively. Four additional constructs, pCB1B::OM*_NSP_*, pOM1B::CB*_NSP_*, pCB1B::OM*_MP_*, and pOM1B::CB*_MP_*, were produced by swapping the *BV1* (encoding NSP) or *BC1* (encoding MP) gene between two ToLCNDV isolates. The primers used for construction are indicated above each genome. (b) *Nicotiana benthamiana*, oriental melon, and cucumber plants after mechanical inoculation with pCB2A + pCB1B, pOM2A + pOM1B, or other infectious clones, as indicated, developed viral symptoms (O) or exhibited no symptoms (X) at 10–12 days post‐inoculation. The viral inoculum used for mechanical sap inoculation was prepared from *N. benthamiana* with symptoms after agroinoculation with an appropriate combination of the clones. Symptoms were observed exclusively in plants inoculated with the constructs carrying the ToLCNDV‐OM *MP* gene. (c) PCR detection of viral DNA in plants after mechanical inoculation with pCB2A + pCB1B (lanes 1, 7, and 13), pCB2A + pCB1B::OM*_NSP_* (lanes 2, 8, and 14), pCB2A + pCB1B::OM*_MP_* (lanes 3, 9, and 15), pOM2A + pOM1B (lanes 4, 10, and 16), pOM2A + pOM1B::CB*_NSP_* (lanes 5, 11, and 17), or pOM2A + pOM1B::CB*_MP_* (lanes 6, 12, and 18). Specific primers were used to amplify the *NSP*, *MP*, or intergenic region (IR) fragment from the OM or CB isolate. Amplicons were obtained only from plants inoculated with the constructs carrying the ToLCNDV‐OM *MP* gene

To determine the roles of NSP and MP in mechanical transmission, two infectious clones, pCB1B and pOM1B, containing a single copy of the DNA‐B genomes of the respective ToLCNDV isolates and 832‐bp  intergenic region (IR) tandem repeats, were generated (Figure 3a). As controls, the three test species were agroinoculated with pCB2A + pCB1B or pOM2A + pOM1B, which resulted in a high disease incidence. Sap extracted from *N. benthamiana* infected with pOM2A + pOM1B, but not with pCB2A + pCB1B, also induced symptoms on the test plants (Table 2 and Figure 3b).

To confirm the role of NSP in mechanical transmission, two infectious clones, pCB1B::OM*_NSP_* and pOM1B::CB*_NSP_*, were generated by exchanging the NSP‐coding genes of two ToLCNDV isolates and tested for infectivity. The agroinoculation of the test plants with pCB2A + pCB1B::OM*_NSP_* or pOM2A + pOM1B::CB*_NSP_* resulted in symptoms on all three plant species (Table 2). Sap extracted from *N. benthamiana* plants infected with pOM2A + pOM1B::CB*_NSP_* mechanically infected all three plant species, and viral DNA was detectable by PCR using gene‐specific primers (Figure 3b,c). However, sap prepared from *N. benthamiana* leaves infected with pCB2A + pCB1B::OM*_NSP_* failed to induce symptoms, and viral DNA was not detected in the test plants after rub inoculation. Thus, a role of NSP in mechanical transmissibility was ruled out in this study.

### 2.4 The ToLCNDV‐OM MP is required for mechanical transmission

To determine the role of the ToLCNDV‐OM movement protein in mechanical transmission, two infectious clones were generated. The pCB1B::OM*_MP_* clone was generated by replacing ToLCNDV‐CB *MP* with ToLCNDV‐OM *MP*. The other clone, designated pOM1B::CB*_MP_*, was generated by replacing ToLCNDV‐OM *MP* with ToLCNDV‐CB *MP*. Agroinoculation of three plant species with pCB2A + pCB1B::OM*_MP_* or pOM2A + pOM1B::CB*_MP_* resulted in symptoms at 7 dpi. Sap extracted from *N. benthamiana* plants infected with pCB2A + pCB1B::OM*_MP_* mechanically infected the test plants (Figure 3b) and viral DNA propagated, as demonstrated by PCR using gene‐specific primers for *NSP*, *MP* or IR (Figure 3c). However, sap prepared from *N. benthamiana* leaves infected with pOM2A + pOM1B::CB*_MP_* failed to induce symptoms, and the virus was not detected after rub inoculation, indicating the involvement of the ToLCNDV‐OM *MP* gene in mechanical transmission.

### 2.5 The 5′ end of the MP of ToLCNDV‐OM is required for mechanical transmissibility

**Figure 4:**
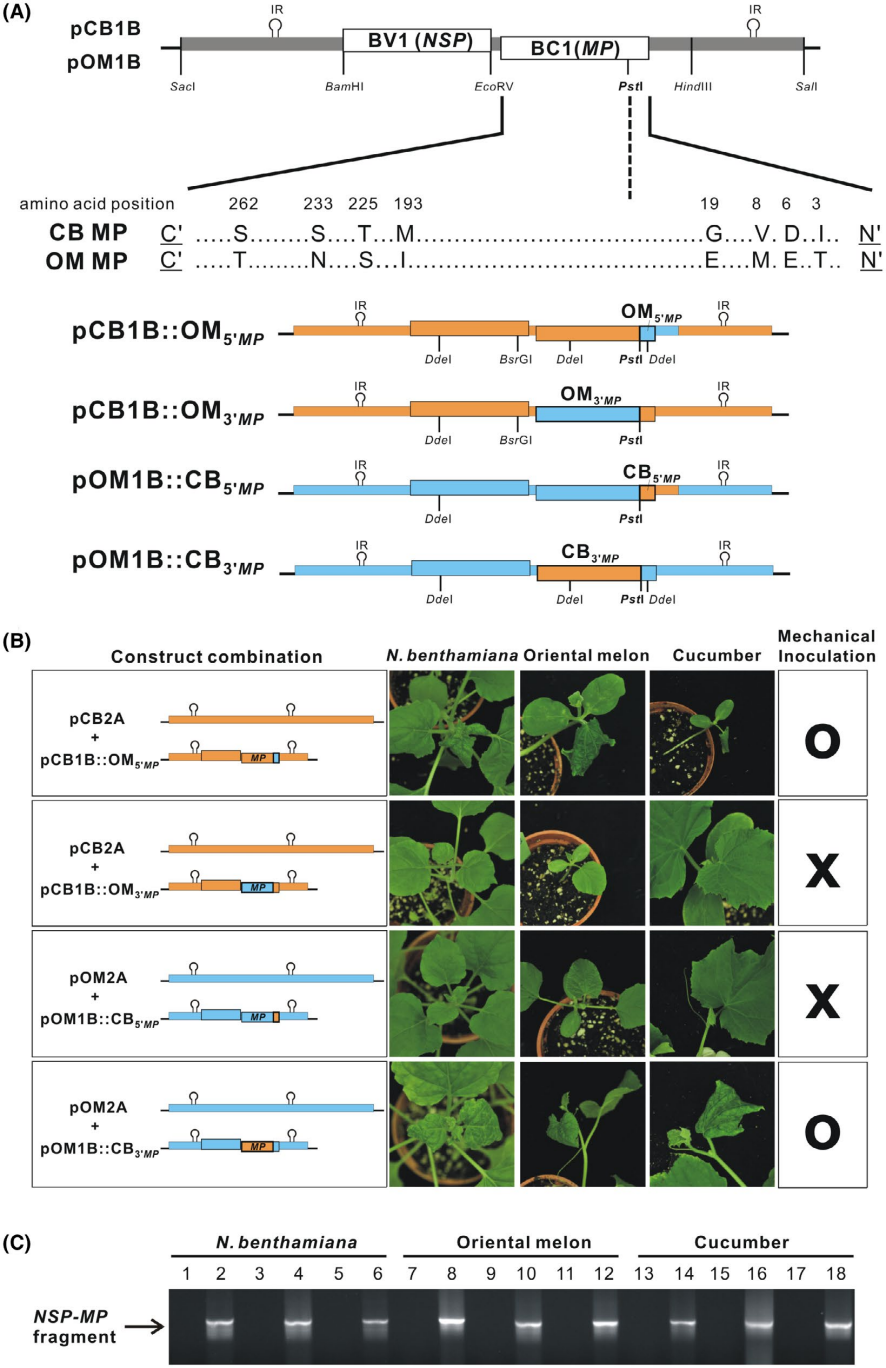
The 5′‐end of the movement protein (MP) of the tomato leaf curl New Delhi virus (ToLCNDV)‐OM is required for mechanical transmissibility. (a) Schematic illustration of the pCB1B and pOM1B infectious clones showing eight different amino acid residues in MP between the ToLCNDV‐OM and CB isolates. Four different amino acids (3rd, 6th, 8th, and 19th) were found at the N terminus, and four other amino acids (193rd, 225th, 233rd, and 262nd) were found at the C terminus of MPs. Two clusters of different amino acids were separated via the *Pst*I restriction enzyme recognition site. Four recombinant constructs, pCB1B::OM_5′_ *_MP_*, pCB1B::OM_3′_ *_MP_*, pOM1B::CB_5′_ *_MP_*, and pOM1B::CB_3′_ *_MP_*, were produced by exchanging the 5′*MP*‐intergenic region (IR) fragment (455 bp) and the 3′‐end *MP* fragment (817 bp) between two isolates using *Eco*RV, *Pst*I, and/or *Hin*dIII cleavage. (b) *Nicotiana benthamiana*, oriental melon, and cucumber plants after mechanical inoculation with the infectious clones, either developed viral symptoms (O) or exhibited no symptoms (X) at 10–12 days post‐inoculation. The viral inoculum used for mechanical sap inoculation was prepared from *N. benthamiana* with symptoms after agroinoculation with an appropriate combination of the clones. Symptoms were observed only in plants inoculated with the constructs carrying the 5′ region of the ToLCNDV‐OM *MP* gene. (c) PCR detection of the *NSP*‐*MP* DNA fragment in plants after mechanical inoculation with pCB2A + pCB1B (lanes 1, 7, and 13), pCB2A + pCB1B::OM_5′_ *_MP_* (lanes 2, 8, and 14), pCB2A + pCB1B::OM_3′_ *_MP_* (lanes 3, 9, and 15), pOM2A + pOM1B (lanes 4, 10, and 16), pOM2A + pOM1B::CB_5′_ *_MP_* (lanes 5, 11, and 17), and pOM2A + pOM1B::CB_3′_ *_MP_* (lanes 6, 12, and 18). Amplicons were obtained only from plants inoculated with the constructs carrying the 5′ end of the *MP* of ToLCNDV‐OM

The comparison of MP amino acid sequences between ToLCNDV‐CB and ToLCNDV‐OM (Figure S2) revealed that the two MPs were highly similar, differing only by four amino acid residues in the N‐terminal region and another four in the C‐terminal region (Figure 4a). To identify the critical amino acid residue(s) involved in mechanical transmissibility, chimeric *MP* genes were constructed through the combination of portions of two viral MP‐encoding genes and tested for infectivity (Figure 4a). The pCB1B::OM_5′_ *_MP_* clone was constructed in the DNA‐B backbone of ToLCNDV‐CB by substituting the 5′ fragment of the *MP* gene with its ToLCNDV‐OM counterpart. pCB1B::OM_3′_ *_MP_* was generated by replacing the 3′ end of ToLCNDV‐CB *MP* with its ToLCNDV‐OM counterpart. Similar approaches were used to construct pOM1B::CB_5′_ *_MP_* and pOM1B::CB_3′_ *_MP_*. Combined with the corresponding DNA‐A infectious clone, all four constructs induced symptoms in *N. benthamiana* after agroinoculation (Table 2).

Mechanical transmission assays using sap extracted from *N. benthamiana* plants infected with pCB2A + pCB1B::OM_5′_ *_MP_* or pOM2A + pOM1B::CB_3′_ *_MP_* resulted in symptoms on *N. benthamiana*, oriental melon, and cucumber (Figure 4b). In contrast, sap from *N. benthamiana* infected with pOM2A + pOM1B::CB_5′_ *_MP_* or pCB2A + pCB1B::OM_3′_ *_MP_* failed to induce symptoms on the test plants after rub inoculation, indicating an important role of the 5′ end of the *MP* of ToLCNDV‐OM in mechanical transmissibility. The presence of chimeric *NSP‐MP* sequences after mechanical inoculation was identified by PCR only in plants inoculated with pCB2A + pCB1B::OM_5′_ *_MP_* or pOM2A + pOM1B::CB_3′_ *_MP_* (Figure 4c). The identity of the *MP* fragments in the mechanically infected *N. benthamiana* was further verified by restriction enzyme digestion and gel analysis. The digestion of *NSP‐MP* amplicons with *Dde*I revealed polymorphisms of the expected sizes from pCB2A + pCB1B::OM_5′_ *_MP_* (271, 431, 643, and 794 bp) and pOM2A + pOM1B::CB_3′_ *_MP_* (233, 431, 643, and 794 bp), which were clearly different from those of pOM2A + pOM1B (233, 431, and 1,437 bp). Because pCB1B::OM_5′_ *_MP_* contained a *BsrG*I recognition site, which was not present in pOM1B::CB_3′_ *_MP_*, *BsrG*I was used to differentiate the two genomes (Figure S3). The results indicate that the 5′ but not the 3′ end of the MP of ToLCNDV‐OM was required for mechanical transmissibility.

### 2.6 A single amino acid in the ToLCNDV‐OM MP determines mechanical transmissibility

**Figure 5:**
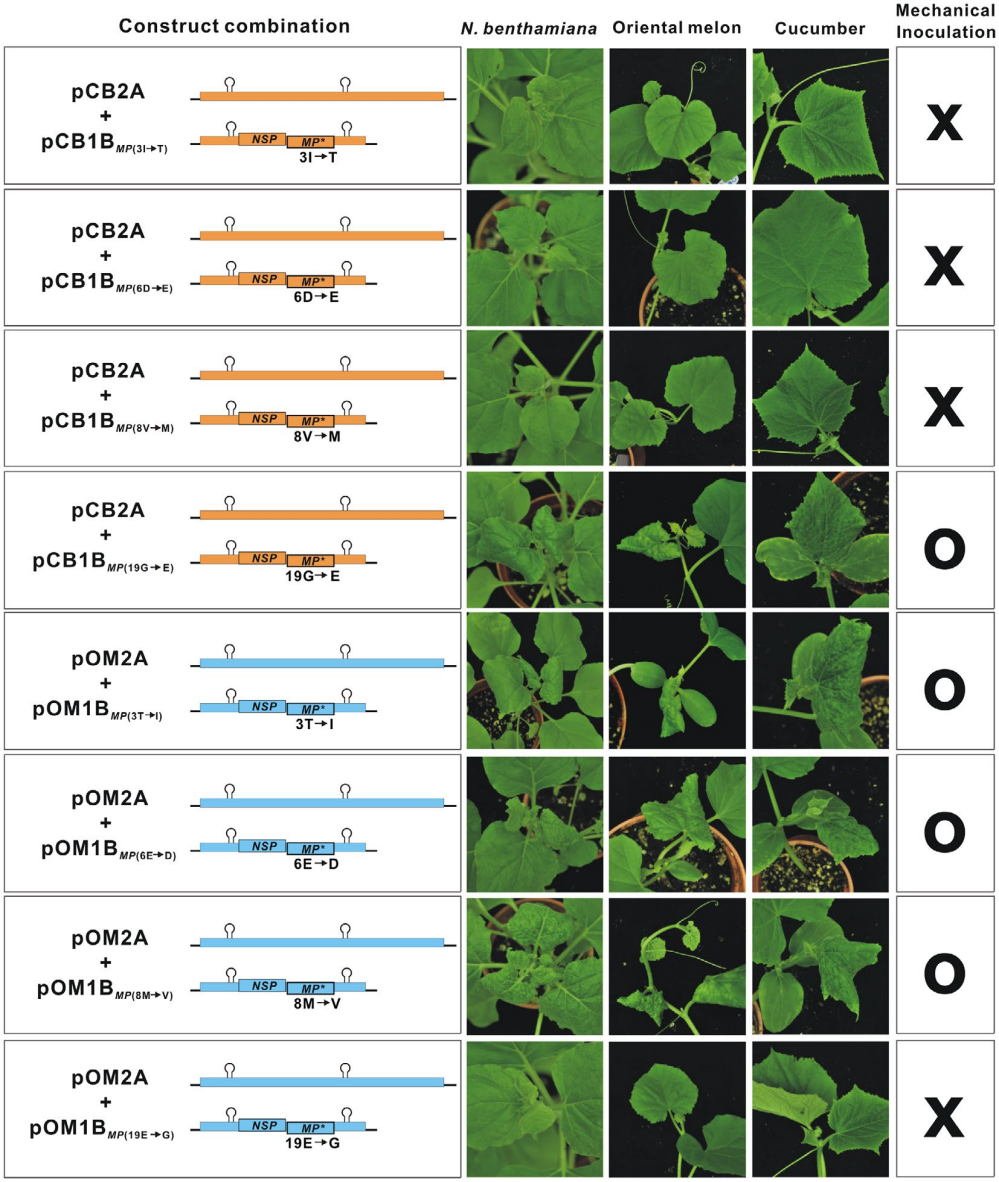
A single glutamate residue in the tomato leaf curl New Delhi virus (ToLCNDV)‐OM movement protein (MP) determines mechanical transmissibility. *Nicotiana benthamiana*, oriental melon, and cucumber plants after mechanical inoculation with pCB2A paired with pCB1B*_MP_*_(3I→T)_, pCB1B*_MP_*_(6D→E)_, or pCB1B*_MP_*_(8V→M)_ carrying a single amino acid substitution at the 3rd, 6th or 8th position of the MP of the CB isolate did not develop visible symptoms (X). However, plants that were mechanically inoculated with pCB2A and pCB1B*_MP_*_(19G→E)_ carrying a substitution mutation at the 19th amino acid, resulting in a change from glycine to glutamate, developed viral symptoms (O). Plants that were mechanically inoculated with pOM2A and pOM1B*_MP_*_(3T→I)_, pOM1B*_MP_*_(6E→D)_, or pOM1B*_MP_*_(8M→V)_ also developed visible symptoms. In contrast, plants that were mechanically inoculated with pOM2A and pOM1B*_MP_*_(19E→G)_, carrying a single amino acid substitution at the 19th amino acid, resulting in a change from glutamate to glycine, failed to develop symptoms. The viral inoculum used for mechanical sap inoculation was prepared from *N. benthamiana* with symptoms after agroinoculation with an appropriate combination of the clones. Abbreviations for amino acids: G, glycine; E, glutamate; I, isoleucine; T, threonine; D, aspartic acid; V, valine; M, methionine

Sequence alignment of ToLCNDV‐OM and ToLCNDV‐CB MPs revealed four amino acid differences (3rd, 6th, 8th, and 19th) in the N‐terminal region (Figure 4a). Site‐directed mutagenesis was performed to identify the specific amino acid residue(s) that are essential for mechanical transmissibility. Eight infectious clones, each containing a specific mutation of these four amino acids, were constructed and tested for infectivity (Figure 5). Agroinoculation and rub inoculation assays revealed that infectious clones pCB1B*_MP_*_(3I→T)_, pCB1B*_MP_*_(6D→E)_, pCB1B*_MP_*_(8V→M)_, pOM1B*_MP_*_(3T→I)_, pOM1B*_MP_*_(6E→D)_, and pOM1B*_MP_*_(8M→V)_, carrying substitution mutations at the 3rd, 6th, and 8th amino acid residues of either CB or OM, did not alter mechanical transmissibility when paired with the corresponding DNA‐A clone. However, ToLCNDV‐CB became mechanically transmissible when the 19th amino acid was changed from glycine (G) to glutamate (E), as demonstrated by the pairing of pCB1B*_MP_*_(19G→E)_ with pCB2A (Figure 5). Moreover, the mechanical transmissibility of ToLCNDV‐OM was abolished by a substitution mutation at the 19th amino acid causing a change from glutamate to glycine, as demonstrated by the pairing of pOM1B*_MP_*_(19E→G)_ with pOM2A. The presence of the viral variants in the mechanically infected plants was confirmed by PCR and sequencing analysis (data not shown).

### 2.7 Nonmechanically transmissible viruses fail to accumulate at detectable levels in plants after sap inoculation

**Figure 6:**
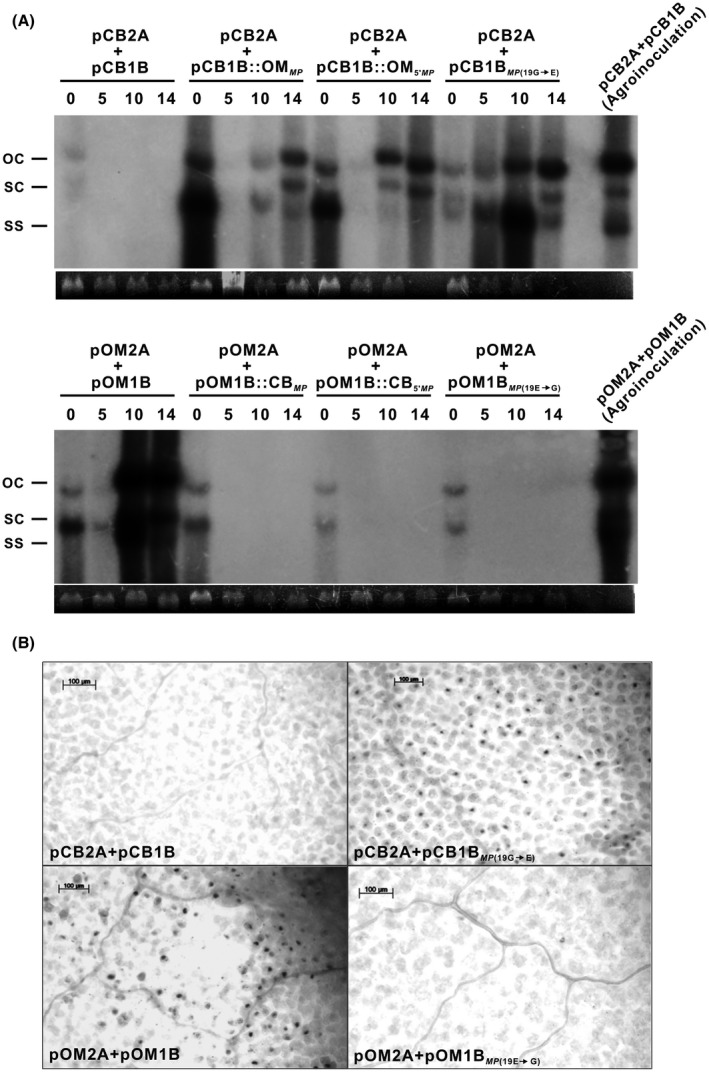
Nonmechanically transmissible viruses fail to accumulate to detectable levels in plants after sap inoculation. (a) Southern blot analysis of viral DNA in the open circular (oc), supercoiled (sc) double‐stranded DNA, and single‐stranded DNA (ss) forms in the cotyledons of oriental melon mechanically inoculated with infectious clones as indicated over time (0, 5, 10, and 14 days post‐inoculation, dpi using the tomato leaf curl New Delhi virus (ToLCNDV)‐OM *MP* probe. DNA prepared from oriental melon inoculated with pCB2A + pCB1B (upper panel) or pOM2A + pOM1B (lower panel) by using *Agrobacterium* was used as a positive control. DNA stained with ethidium bromide was used as the loading control. (b) In situ hybridization of *Nicotiana benthamiana* leaves with the ToLCNDV‐OM MP probe at 5 dpi with the infectious clones, as indicated. The dark spots observed in samples inoculated with pCB2A + p pCB1B*_MP_*_(19G→E)_ and pOM2A + pOM1B represent viral hybridization signals. Bar = 100 μm. Only representative examples are shown

Oriental melon plants that were inoculated with ToLCNDV infectious clones developed visible symptoms at 7–10 dpi. After mechanical transmission, Southern blot analysis was performed to determine the viral titres present in oriental melon cotyledons at 0, 5, 10, and 14 dpi using a ToLCNDV‐OM *MP* probe (Figure 6a). Viral DNA in the form of open circular (oc) and supercoiled (sc) double‐stranded DNA as well as single‐stranded DNA (ss) could be detected in all plants immediately after sap inoculation (0 dpi). Surprisingly, viral DNA was barely detectable or undetectable at 5 dpi in all oriental melon plants except those inoculated with pCB2A + pCB1B*_MP_*_(19G→E)_. At 10 and 14 dpi, viral DNA could be detected in all plants inoculated with infectious clones pOM2A + pOM1B, pCB2A + pCB1B::OM*_MP_*, pCB2A + pCB1B::OM_5′_ *_MP_*, and pCB2A + pCB1B*_MP_*_(19G→E)_, which have been shown to be mechanically transmittable. However, no viral DNA was detected in plants inoculated with clones pCB2A + pCB1B, pOM2A + pOM1B::CB*_MP_*, pOM2A + pOM1B::CB_5′_ *_MP_*, and pOM2A + pOM1B*_MP_*_(19E→G)_, which were not mechanically transmissible. PCR analysis of samples prepared from apical leaves also identified the expected amplicons only in plants inoculated with the infectious constructs that were mechanically transmissible (data not shown). Further analysis via in situ hybridization identified ToLCNDV‐OM viruses in *N. benthamiana* leaves inoculated with pOM2A + pOM1B or pCB2A + pCB1B*_MP_*_(19G→E)_ at 5 dpi. No signal was detected in leaf samples inoculated with pCB2A + pCB1B or pOM2A + pOM1B*_MP_*_(19E→G)_ (Figure 6b). The results indicate the critical role of glutamate as the 19th residue of MP in the mechanical transmissibility of the ToLCNDV OM isolate.

### 2.8 The glutamate residue is related to the tertiary structure of the ToLCNDV‐OM MP

**Figure 7:**
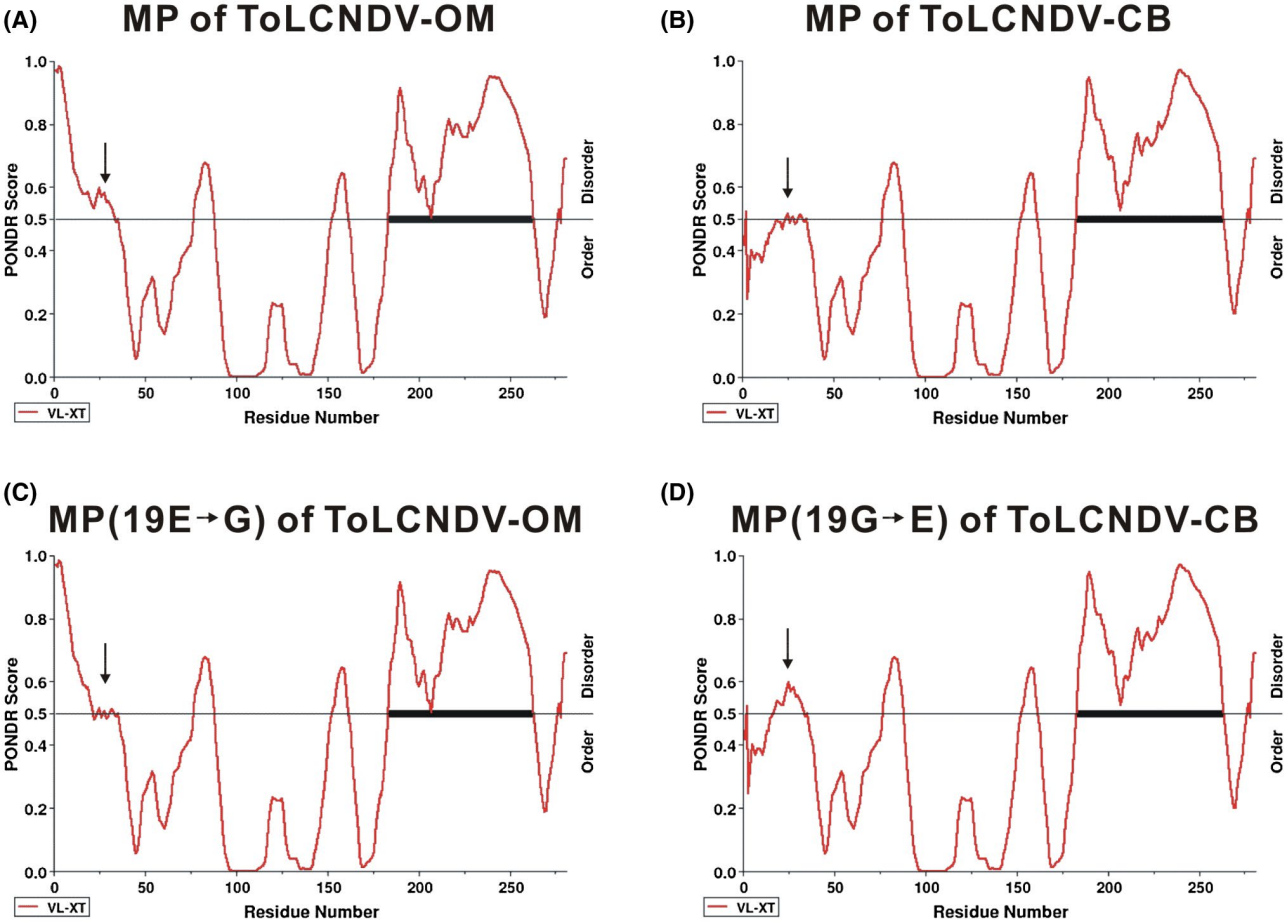
Prediction of natural disordered regions in movement proteins (MPs) using VL‐XT PONDR software. PONDR plot of the disorder score for each of the MP amino acid residue of the tomato leaf curl New Delhi virus (ToLCNDV)‐OM (a), ToLCNDV‐CB (b), ToLCNDV‐OM*_MP_*_(19E→G)_ (c), and ToLCNDV‐CB*_MP_*_(19G→E)_ (d). The horizontal lines indicate the threshold for disorder prediction. The 19th amino acid is indicated by an arrow

Protein sequence analysis using the predictor of natural disordered regions (PONDR) revealed that the N terminus of the MP of ToLCNDV‐OM was probably a disordered region and that the counterpart in ToLCNDV‐CB was an ordered region (Figure 7). In contrast, the deduced pOM1B*_MP_*_(19E→G)_ amino acid sequence (ToLCNDV‐OM mutant), which differed only at the 19th (glycine, G) residue from that of ToLCNDV‐OM, was an ordered region. The deduced pCB1B*_MP_*_(19G→E)_ amino acid sequence (ToLCNDV‐CB mutant), containing glutamate (E) instead of glycine at the 19th position, was a disordered region. The results suggest a close relationship between the 19th glutamate and the tertiary structure of MP in the ToLCNDV OM isolate. However, sequence alignment of MP sequences available in the database revealed that glutamate as the 19th residue was not conserved among ToLCNDV isolates. Both the ToLCNDV‐severe isolate (nonmechanically transmissible) and the ToLCNDV‐potato isolate (mechanically transmissible) exhibited glutamate as the 19th amino acid residue, indicating that glutamate alone is not sufficient to determine mechanical transmissibility.

## 3 DISCUSSION

Begomoviruses are often transmitted by whiteflies and are rarely mechanically transmissible by rub or sap inoculation (Wege and Pohl, 2007). Only a few known species in the *Begomovirus* genus have been reported to be mechanically transmissible to their natural hosts (Gilbertson *et al.*, 1991; Usharani *et al.*, 2004; Tsai *et al.*, 2011). ToLCNDV is a begomovirus with a bipartite (DNA‐A and DNA‐B) genome (Padidam *et al.*, 1995). In the present study, genetic and pathological approaches were applied to identify the molecular determinants involved in mechanical transmissibility in a ToLCNDV‐OM isolate originally collected from a diseased oriental melon. Unlike ToLCNDV‐OM, the closely related ToLCNDV‐CB isolate is not mechanically transmissible to oriental melon, cucumber or *N. benthamiana* via sap inoculation. The two ToLCNDV isolates show strong nucleotide sequence identity for both DNA‐A (96.5%) and DNA‐B (92.2%), and exhibit very similar host ranges. Genetic evidence indicates that DNA‐B of ToLCNDV‐OM plays a critical role in mechanical transmissibility, consistent with findings for TYLCTHV and abutilon mosaic virus (AbMV) (Wege and Pohl, 2007; Tsai *et al.*, 2011).

DNA‐B contains both the *NSP* and *MP* genes. *NSP* encodes a protein that is presumably responsible for the transportation of single‐stranded or double‐stranded DNA between the cytoplasm and the nucleus. *MP* encodes a protein required for cell‐to‐cell movement. Although *NSP* has been reported to be required for begomovirus virulence (Hussain *et al.*, 2005; Zhou *et al.*, 2007), a role of this gene in mechanical transmissibility was ruled out by testing the infectious clone pOM1B::*NSP*_ins39_ in the present study. This clone carries an extra 39‐nt fragment from a nonmechanically transmissible isolate (ToLCNDV‐severe) at the 5′ end of ToLCNDV‐OM *NSP*, and together with pOM2A it failed to infect *N. benthamiana* and oriental melon via sap inoculation. Further substitution of *NSP* between ToLCNDV‐OM and ToLCNDV‐CB also ruled out the possible involvement of *NSP* in mechanical transmission because swapping *NSP* between these two isolates did not alter the mode of transmissibility.

Genetic evidence derived from a series of mutations has revealed that MP in DNA‐B contains the key determinant required for mechanical transmission in ToLCNDV‐OM. The exchange of MPs between ToLCNDV‐OM and ToLCNDV‐CB revealed that an infectious clone carrying ToLCNDV‐OM MP, but not ToLCNDV‐CB MP, was able to induce symptoms on three test plant species after sap inoculation. Moreover, it appears that the N terminus of DNA‐B MP contains the elements required for mechanical transmissibility. Sequence alignment revealed that ToLCNDV‐OM and ToLCNDV‐CB MPs share 97.2% similarity, differing in only eight amino acids, four of which are located in the N terminus, while the other four are located at the C terminus. The involvement of the C terminus was ruled out by DNA‐B swapping assays. The evaluation of a series of point mutations revealed that glutamate at the 19th amino acid position at the N‐terminal end of MP is critical for the mechanical transmission of ToLCNDV‐OM. Point mutations affecting the 3rd, 6th, or 8th amino acid residue of ToLCNDV‐CB or ToLCNDV‐OM did not affect the mechanical transmissibility of either isolate. Moreover, ToLCNDV‐CB, which is not mechanically transmissible, became mechanically transmissible when a point mutation at the 19th amino acid of MP caused a change from glycine (G) to glutamate (E). In contrast, changing the 19th amino acid from glutamate to glycine in ToLCNDV‐OM rendered the virus nonmechanically transmissible, which might be due to the characteristics of the amino acids, as glycine is nonpolar and glutamate is negatively charged.

Although the glutamate residue at the 19th position of the ToLCNDV‐OM MP plays a critical role in mechanical transmission, glutamate alone is seemingly not sufficient to determine mechanical transmissibility. Sequence alignment revealed that the MPs of both the ToLCNDV‐severe (nonmechanically transmissible) and ToLCNDV‐potato (mechanically transmissible) isolates also exhibited glutamate at the 19th position, suggesting the presence of other factors or determinants that may contribute to the mechanical transmission of ToLCNDV‐OM. This assumption is supported by findings obtained using PONDR VL‐XT. PONDR plots can be used to visualize regions within disordered regions and to determine potential binding sites in proteins (Oldfield *et al.*, 2005; Cheng *et al.*, 2007). Disordered protein regions, characterized by the lack of a fixed tertiary structure, have been proposed to be involved in many biochemical functions, including protein binding and recognition (Dunker *et al.*, 2002; Xue *et al.*, 2010). PONDR analyses suggested that the N terminus of ToLCNDV‐OM MP is a disordered region; a switch from glutamate to glycine at the 19th amino acid position changes the prediction to an ordered region. Moreover, the N terminus of ToLCNDV‐CB MP is predicted to be an ordered region; however, the sequence is predicted to be a disordered region after the substitution of glycine with glutamate at the 19th amino acid.

In addition to MP, CP may be involved in mechanical transmissibility in ToLCNDV‐OM as demonstrated in BGMV, another member of the *Begomovirus* genus. The BGMV CP has been demonstrated to be required for both mechanical and whitefly transmissions (Azzam *et al.*, 1994). A BGMV mutant with a defective CP fails to induce symptoms on sap inoculation, but an infection develops after the mutant is biolistically bombarded into leaf cells because biolistic inoculation can deliver the virus directly into the host nucleus. Thus, CP is required for the localization of BGMV to the nucleus. Intriguingly, the CP of two geminiviruses, mungbean yellow mosaic virus (MYMV) and ACMV, have been reported to contain a nuclear localization signal (NLS) (Unseld *et al.*, 2001; Guerra‐Peraza *et al.*, 2005), suggesting the involvement of CP in nuclear localization. Therefore, the difference in mechanical transmissibility between ToLCNDV‐OM and ToLCNDV‐CB may result from the interactions of CP and MP, which was shown not to be required for the mechanical transmission of ToLCNDV in the present study.

MP has also been implicated in intra‐ and intercellular trafficking by interacting with host proteins (Jeske, 2009), which could lead to different modes of transmission as seen in different ToLCNDV isolates. AbMV MP can interact with host kinases and affect the development of symptoms and the accumulation of viral DNA in host cells (Kleinow *et al.*, 2009). Studies have identified histone H3 (Zhou *et al.*, 2011), synaptotagmin (SYTA) (Lewis and Lazarowitz, 2010), and the 70 kDa heat shock protein cpHSC70‐1 (Krenz *et al.*, 2010) as proteins that can interact with the MP of begomoviruses. Histone H3 is probably required for the assembly of the MP‐nucleoprotein complex in the nucleus. SYTA is required for cell‐to‐cell trafficking and systemic infection. The cpHSC70‐1 protein regulates the MP conformation at the cell periphery and localization to chloroplasts.

Viral MPs are required for both cell‐to‐cell and long‐distance movements. Studies on AbMV, another geminivirus, have suggested that a so‐called “pilot domain” (amino acid residues from 1 to 49) of MP is responsible for the transportation of the virus to cell periphery or nucleus (Zhang *et al.*, 2002). Two models have been proposed to explain the functions of MP in geminiviruses (Rojas *et al.*, 2005; Jeske, 2009). For phloem‐limited begomoviruses such as AbMV and cabbage leaf curl virus (CaLCuV), MP is thought to attach to the NSP‐bound viral DNA and form a complex that localizes along the plasma membrane or is transferred to adjacent cells through plasmodesmata. For mesophyll‐invading begomoviruses, MP is thought to take over viral DNA from NSP after being exported from the nucleus to the cytoplasm and deliver it to adjacent cells. Most nonmechanically transmissible begomoviruses have been reported to be phloem limited because they can be introduced into the phloem translocation stream through the stylets of insects and become systemic (Czosnek *et al.*, 2017). One of the reasons that ToLCNDV‐CB fails to be mechanically transmissible and to initiate systemic infection after sap inoculation may be its inability to travel beyond the site of inoculation. ToLCNDV‐CB is probably a phloem‐limited virus because this isolate is unable to propagate in the mesophyll after sap inoculation, as demonstrated by Southern blot and in situ hybridization analyses. In contrast, ToLCNDV‐OM is capable of replicating in mesophyll after sap inoculation.

For systemic infection, ToLCNDV must be loaded into the phloem sieve tube system for long‐distance movement. ToLCNDV‐OM is able to propagate and travel through mesophyll cells, the bundle sheath, and eventually the phloem after sap inoculation, which is very rare for a geminivirus (Wang *et al.*, 1996). In contrast, ToLCNDV‐CB apparently has to rely on the stylet of its insect vector to reach the phloem. After sap inoculation, ToLCNDV‐CB is unable to move beyond its inoculation foci, for which there may be many possible explanations. ToLCNDV‐CB may trigger plant defence, fail to manipulate plasmodesmata, and be unable to replicate. A time‐course study of viral accumulation by Southern blotting (Figure 6) revealed that ToLCNDV‐OM DNA is barely detectable or undetectable at 5 dpi in oriental melon plants and can be detected again at 10 and 14 dpi, suggesting the suppression of plant defence during the early stages of virus–plant interactions. ToLCNDV‐OM, but not ToLCNDV‐CB, might be able to overcome host defence reactions and, thus, infect the host systemically after sap inoculation.

In conclusion, genetic experiments have clearly demonstrated that the glutamate residue at the 19th position in the N terminus of the MP of the ToLCNDV‐OM DNA‐B genome is required for mechanical transmission. Our genetic evidence also ruled out the involvement of NSP in mechanical transmission. It is very likely that MP interacts with host proteins to enable the mechanical transmission of ToLCNDV‐OM. Understanding the molecular mechanisms involved in the mechanical transmission of viruses could contribute to the development of more efficient strategies to manage diseases caused by begomoviruses.

## 4 EXPERIMENTAL PROCEDURES

### 4.1 Characteristics and origins of ToLCNDV isolates

ToLCNDV‐OM was originally isolated from a diseased oriental melon (*Cucumis melo* “Silver Light”) collected from Yilan, Taiwan in 2007 (Chang *et al.*, 2010). ToLCNDV‐CB (accession numbers MK883715 and MK883716) was originally isolated from a diseased cucumber in Thailand. The sequences of ToLCNDV‐OM (accession numbers GU180095 and MK883714), ToLCNDV‐severe (accession numbers U15015 and U15017) and ToLCNDV‐potato (accession numbers AY286316 and AY158080) were retrieved from the GenBank database of NCBI. Sequence alignment was conducted with the ClustalW algorithm of the MegAlign program available in Lasergene 7 software (DNASTAR).

### 4.2 Construction of infectious viral DNAs

Viral infectious clones of ToLCNDV‐OM and ToLCNDV‐CB were constructed via the rolling circle amplification (RCA) method (Wu *et al.*, 2008). The infectious clones pOM2A (formerly pGPhi‐ToLCNDV‐2A) and pOM2B (formerly pGPhi‐ToLCNDV‐2B) containing tandem repeated sequences of ToLCNDV‐OM DNA‐A and DNA‐B, respectively, were constructed in the binary vector pGANP (Lin *et al.*, 2011) in a previous study (Chang *et al.*, 2010). The infectious clones, pCB2A and pCB2B of ToLCNDV‐CB, were constructed in the binary vector pCAMBIA1304 (Cambia). The oligonucleotide primers (FJJ2007‐13 and FJJ2007‐15) used for the construction of infectious clones are listed in Table S2.

A 39‐nt fragment was amplified by PCR using the primers FJJ2009‐26, FJJ2009‐27, and FJJ2009‐32, corresponding to sequences from the *NSP* gene of the ToLCNDV‐severe isolate (Hussain *et al.*, 2005). The amplicon was fused with the ToLCNDV‐OM DNA‐B fragment digested with *Nco*I and *Nhe*I, and the resultant fragment was cloned into *Nco*I and *Nhe*I‐digested pGANP to create pOM2B::*NSP*_ins39_.

An *NSP*‐*MP* fragment was amplified either from pOM2B, with the primers FJJ2010‐99 and FJJ2010‐100 for *NSP* or FJJ2010‐101 and FJJ2010‐102 for *MP*, or from pCB2B, with the primers FJJ2010‐98 and FJJ2010‐100 for *NSP* and FJJ2010‐101 and FJJ2010‐102 for *MP* (Figure 3a and Table S2). The IR fragment was amplified from either pOM2B, with the primers FJJ2010‐95, FJJ2010‐97, FJJ2010‐103, and FJJ2010‐104, or from pCB2B, with the primers FJJ2010‐95, FJJ2010‐96, FJJ2010‐103, and FJJ2010‐104 (Figure 3 and Table S2). A recognition site for restriction endonuclease (*Sac*I, *Bam*HI, *Eco*RV, *Hin*dIII or *Sal*I) was incorporated into the primer as appropriate to facilitate cloning. *NSP*‐*MP* and IR were fused to form an IR‐*NSP*‐*MP*‐IR fragment. The resultant fragment was digested with *Sac*I and *Sal*I and cloned into pCAMBIA1304 to generate pOM1B or pCB1B.

### 4.3 Recombination of partial 5′ or 3′ *MP* sequences between ToLCNDV‐CB and ToLCNDV‐OM

Four infectious clones, each containing a chimeric MP, were constructed in the backbone of pOM1B or pCB1B. The *Hin*dIII‐*Pst*I fragment (partial IR and 5′ end MP, 455 bp) of pCB1B was replaced with its counterpart from pOM1B to generate pCB1B::OM_5′_ *_MP_*. The *Pst*I‐*Eco*RV fragment (3′ end MP, 817 bp) of pCB1B was replaced with its counterpart from pOM1B to generate pCB1B::OM_3′_ *_MP_*. Similarly, the *Hin*dIII‐*Pst*I fragment and *Pst*I‐*Eco*RV of pOM1B were replaced with the counterparts from pCB1B to create pOM1B::CB_5′_ *_MP_* and pOM1B::CB_3′_ *_MP_*, respectively.

### 4.4 Single amino acid substitutions in MP

Point mutations at each of four amino acid residues (3rd, 6th, 8th, and 19th) of MP (Figure 4a) were individually generated in pCB1B and pOM1B using a QuickChange XL Site‐Directed Mutagenesis Kit following the manufacturer's recommendations (Stratagene). In total, eight clones (pCB1B*_MP_*_(3I→T)_, pCB1B*_MP_*_(6D→E)_, pCB1B*_MP_*_(8V→M)_, pCB1B*_MP_*_(19G→E),_ pOM1B*_MP_*_(3T→I)_, pOM1B*_MP_*_(6E→D)_, pOM1B*_MP_*_(8M→V)_, and pOM1B*_MP_*_(19E→G)_) were generated.

### 4.5 Agroinoculation and mechanical inoculation

Infectious clones were individually transformed into *Agrobacterium tumefaciens* C58 by using a GenePulser II electroporator (Bio‐Rad). Agroinoculation was conducted according to the procedure described by Llave *et al. *(2000) with some modifications. Briefly, 0.5 ml of an overnight bacterial culture was recultured in 10 ml of Luria Bertani (LB) medium (pH 5.6) containing kanamycin (50 μg/ml), streptomycin (50 μg/ml), 2‐*N*‐morpholino‐ethanesulfonic acid (MES, 10 mM), and acetosyringone (AS, 0.04 mM) at 28 °C for 16 hr on a shaker set at 200 rpm. The bacterial cells were pelleted by centrifugation at 5,000 × g for 10 min and resuspended in 10 ml of an infiltration solution (10 mM MgCl_2_, 0.15 mM AS, pH 5.6). Agrobacteria carrying the infectious DNA‐A or DNA‐B constructs were co‐injected in equal amounts into the leaves of *N. benthamiana*, oriental melon (*C. melo* “Silver Light”) or cucumber (*C. sativus* “Vantage”). After agroinoculation, *N. benthamiana* leaves with symptoms (c.1 g) were collected and ground in 0.01 M potassium phosphate buffer (pH 7) (1:20, wt/vol). The resultant sap was inoculated onto *N. benthamiana* leaves or cotyledon explants of oriental melon and cucumber plants by rubbing with carborundum powder. All inoculated plants were kept in a greenhouse at 25–28 °C to observe symptom development.

### 4.6 Detection of viral DNA by PCR

DNA was extracted from plant leaves following the method described by Lin *et al. *(2011). PCR was performed in a 25 μl reaction containing 100 ng DNA, 20 each of the primers at nM, 0.05 mM dNTPs, 0.25 U Pro *Taq* Plus DNA polymerase (Protech Technology) and buffer. The conditions used for amplification were as follows: one cycle at 95 °C for 5 min followed by 35 cycles of 95 °C for 1 min, 50–67 °C depending on the specific primer pairs used (Table S1) for 1 min and 72 °C for 1 min, with a final cycle at 72 °C for 10 min. The PCR products were subjected to electrophoresis in a 0.8% agarose gel. Sequencing was performed using an ABI PRISM 3730 automatic DNA sequencer (Applied Biosystems) available at the Biotechnology Center, National Chung Hsing University (Taichung, Taiwan).

### 4.7 Southern blot hybridization

After mechanical inoculation, the cotyledons of oriental melon were harvested at 0, 5, 10, and 14 dpi and subjected to DNA isolation. DNA (5 μg) was subjected to electrophoresis in a 1% agarose gel and transferred to a nylon membrane (PerkinElmer) via the alkaline transfer method, and hybridization was performed on the full‐length *MP* of ToLCNDV‐OM. The probe was labelled by α‐[^32^P]dATP. The hybridization and detection of the probe were performed according to the methods described by Kon *et al. *(2003). DNA isolated from the apical leaves of oriental melon agroinoculated with ToLCNDV‐OM (pOM2A + pOM1B) or ToLCNDV‐CB (pCB2A + pCB1B) was used as a positive control.

### 4.8 In situ hybridization

Tissue fixation and in situ hybridization were performed following the protocol described by Takeda *et al. *(2011) with some modifications. *N. benthamiana* leaves at 5 dpi were collected, cut into small pieces, and fixed in solution containing 50% ethanol, 5% acetic acid, and 5% formaldehyde (FAA) for 2 hr at room temperature. Leaf samples were soaked in 30% ethanol for 20 min and then in sterile water twice for 20 min each. The samples were soaked in Tris‐EDTA (TE) buffer (100 mM Tris‐HCl, 50 mM EDTA, pH 8.0) for 30 min and treated with 2 μg/ml proteinase K for 30 min at 37 °C. The samples were next washed with phosphate‐buffered saline (PBS) (140 mM NaCl, 2.7 mM KCl, 10 mM Na_2_HPO_4_, and 1.8 mM KH_2_PO_4_, pH 7.4) and fixed in 4% paraformaldehyde for 20 min at 4 °C. The samples were washed with PBS again and incubated in hybridization solution for 20 min. The DNA probe was labelled and detected using a Digoxigenin (DIG)‐High Prime DNA Labeling and Detection Starter Kit I (Roche). The samples were incubated in hybridization solution containing a DIG‐labelled *MP* probe (200 bp) at 50 °C overnight. The detection of the probe was performed via an immunological assay using alkaline phosphatase‐conjugated DIG antibodies followed by nitroblue tetrazolium and 5‐bromo‐4‐chloro‐3‐indolyl phosphate (NBT/BCIP) colour development. Samples were photographed under a Zeiss light microscope equipped with AxioVision Rel 4.8 software (Carl Zeiss).

### 4.9 Predictor of natural disordered regions

The PONDR within the movement protein sequences of ToLCNDV‐OM and ToLCNDV‐CB was performed by using the PONDR VL‐XT predictor available at http://www.pondr.com/.

## Supporting information


**FIGURE S1** Symptoms of *Nicotiana benthamiana*, oriental melon, and cucumber plants after agroinoculation with the infectious clones at 10–12 days post‐inoculation

**FIGURE S2** Alignment of the amino acid residues of the movement proteins of the tomato leaf curl New Delhi virus (ToLCNDV)‐CB and ToLCNDV‐OM

**FIGURE S3** Confirmation of the presence of the 5′ or 3′ *MP* fragment in *Nicotiana benthamiana*, oriental melon, and cucumber plants after mechanical inoculation with the tomato leaf curl New Delhi virus (ToLCNDV)‐CB or ToLCNDV‐OM mutant

**TABLE S1** Characteristics of oligonucleotide primers used to amplify the viral DNA of the tomato leaf curl New Delhi virus (ToLCNDV)‐CB and ToLCNDV‐OM isolates in this study

**TABLE S2** Characteristics of the oligonucleotide primers used to construct infectious clones of tomato leaf curl New Delhi virus

## Data Availability

The data that support the findings of this study are available from the corresponding author upon reasonable request.
