# Supplementary material for: A single amino acid substitution in the movement protein enables the mechanical transmission of a geminivirus
Source: Mol Plant Pathol. 2020 Feb 20;21(4):571–88. doi: 10.1111/mpp.12917 (PMC7060137; doi:10.1111/mpp.12917)
Supplement: Supplementary file 1 — FIGURE S1 Symptoms of Nicotiana benthamiana, oriental melon, and cucumber plants after agroinoculation with the infectious clones at 10–12 days post‐inoculation [file MPP-21-571-s001.docx]

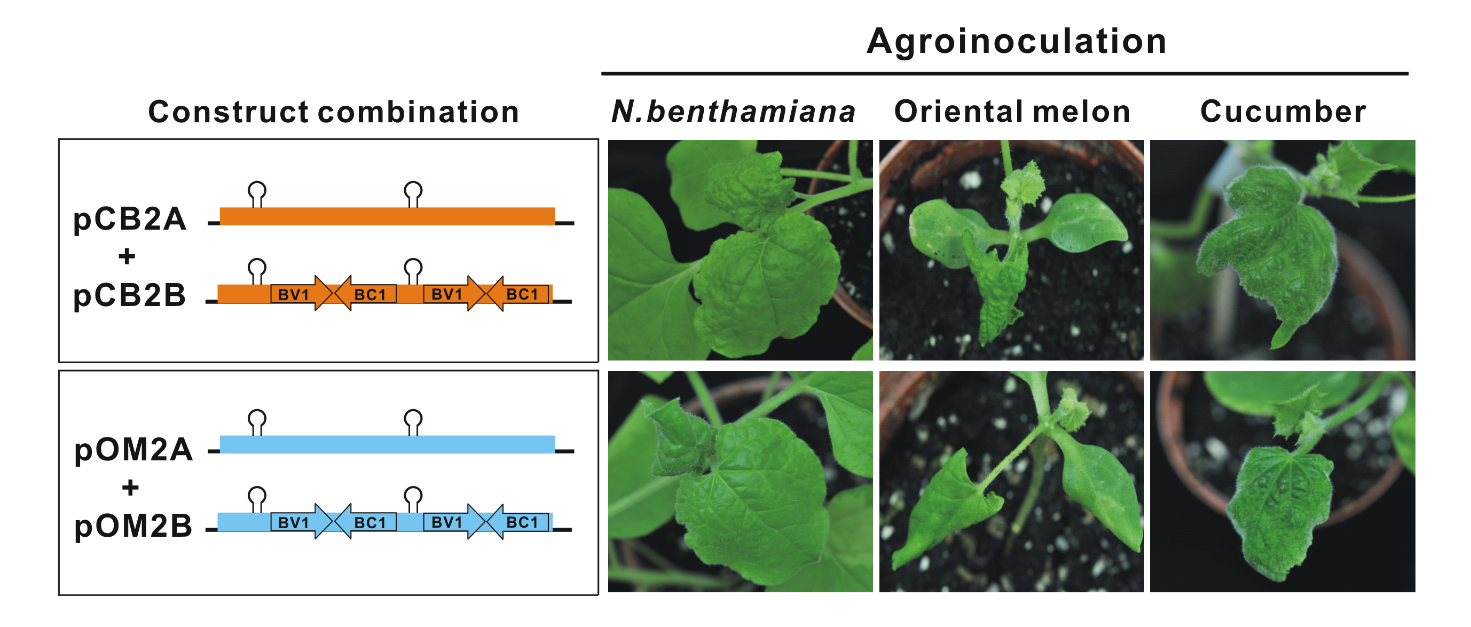


**Fig. S1.** Symptoms of *N. benthamiana*, oriental melon, and cucumber plants after agroinoculation with the infectious clones at 10 to 12 dpi.
