# Supplementary material for: A single amino acid substitution in the movement protein enables the mechanical transmission of a geminivirus
Source: Mol Plant Pathol. 2020 Feb 20;21(4):571–88. doi: 10.1111/mpp.12917 (PMC7060137; doi:10.1111/mpp.12917)
Supplement: Supplementary file 2 — FIGURE S2 Alignment of the amino acid residues of the movement proteins of the tomato leaf curl New Delhi virus (ToLCNDV)‐CB and ToLCNDV‐OM [file MPP-21-571-s002.docx]

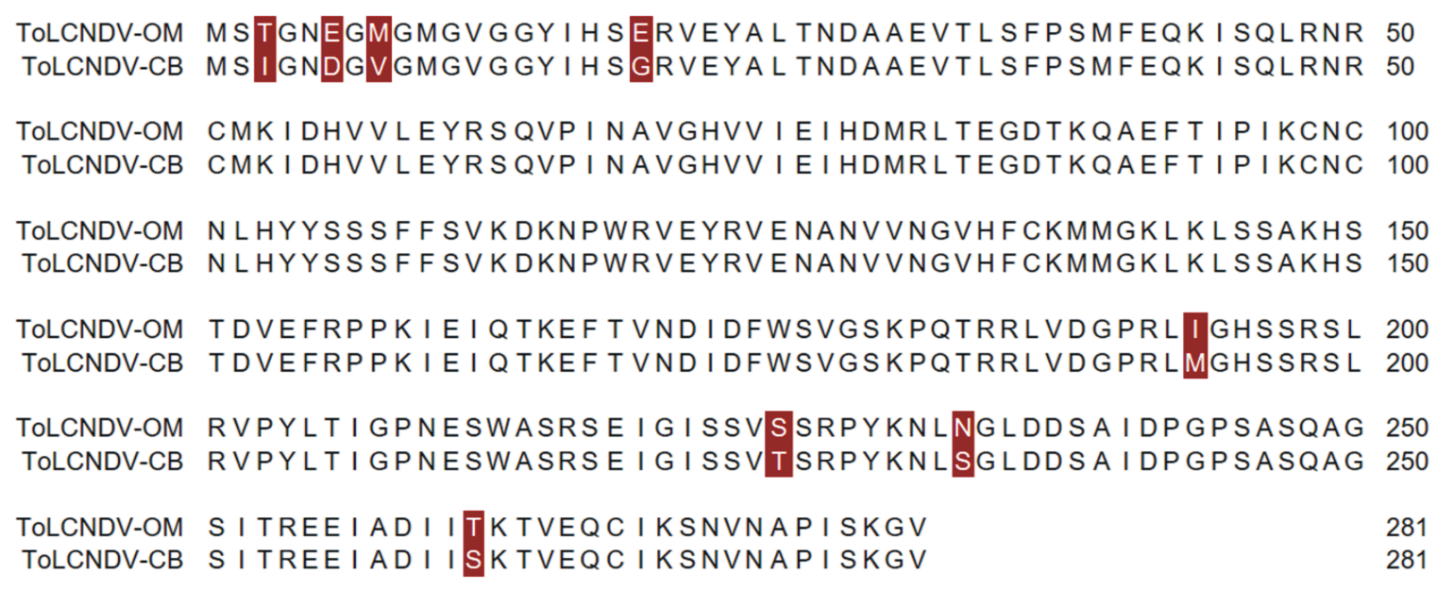


**Fig. S2.** Alignment of the amino acid residues of the movement proteins of the tomato leaf curl New Delhi virus (ToLCNDV)-CB and ToLCNDV-OM, revealing eight different amino acid residues at 3^rd^, 6^th^, 8^th^, 19^th^, 193^rd^, 225^th^, 233^rd^ and 262^nd^ (indicated by red boxes).
