# Supplementary material for: A single amino acid substitution in the movement protein enables the mechanical transmission of a geminivirus
Source: Mol Plant Pathol. 2020 Feb 20;21(4):571–88. doi: 10.1111/mpp.12917 (PMC7060137; doi:10.1111/mpp.12917)
Supplement: Supplementary file 3 — FIGURE S3 Confirmation of the presence of the 5′ or 3′ MP fragment in Nicotiana benthamiana, oriental melon, and cucumber plants after mechanical inoculation with the tomato leaf curl New Delhi virus (ToLCNDV)‐CB or ToLCNDV‐OM mutant [file MPP-21-571-s003.docx]

**
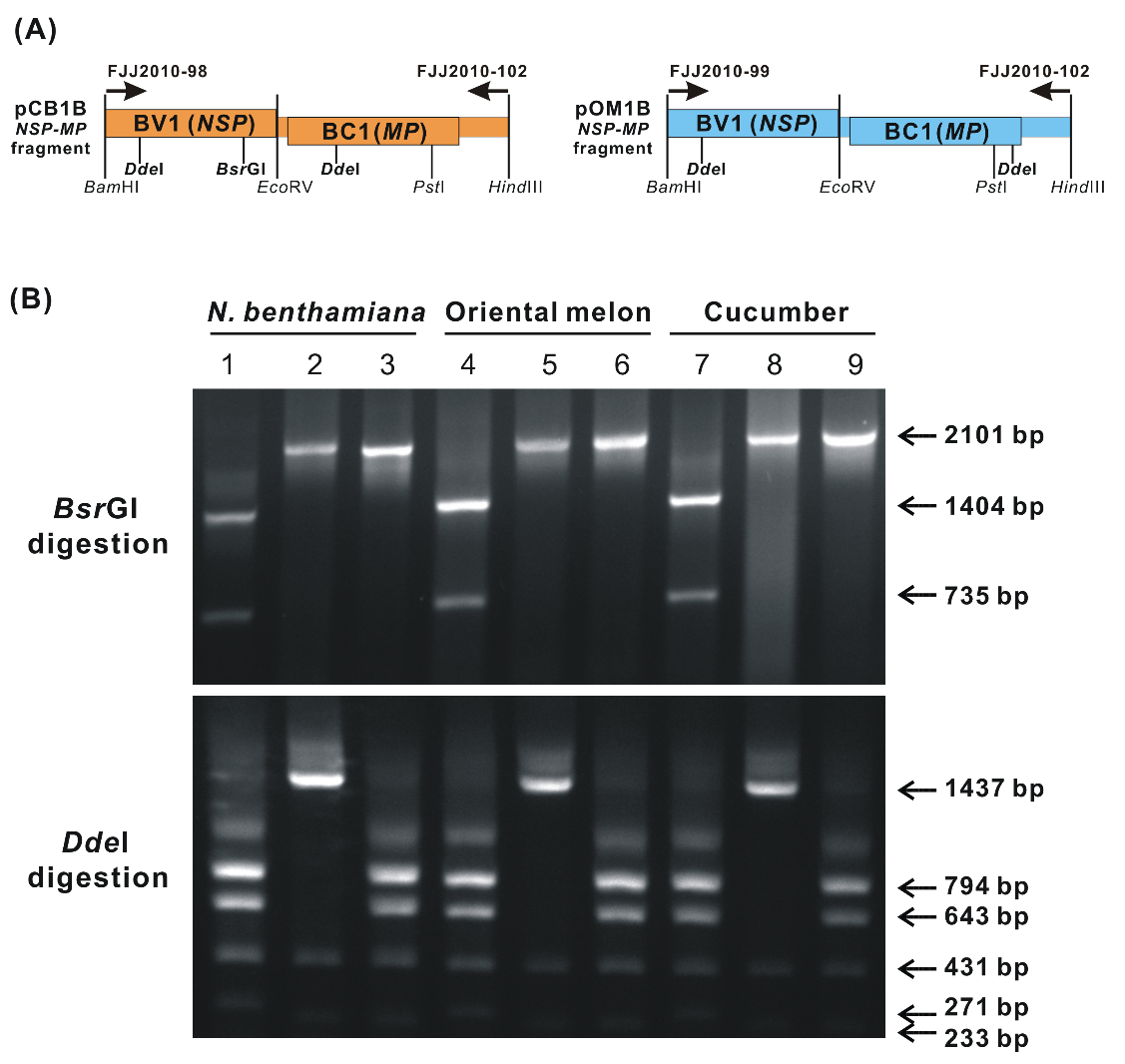
**

**Fig. S3.** Confirmation of the presence of the 5' or 3' *MP* fragment in *N. benthamiana*, oriental melon, and cucumber plants after mechanical inoculation with the tomato leaf curl New Delhi virus (ToLCNDV)-CB or ToLCNDV-OM mutant. (A) Restriction maps of the *NSP*-*MP* fragments amplified from pCB1B and pOM1B with the primers as indicated. (B) Restriction enzyme digestion of the *NSP-MP* fragments amplified from plants inoculated with pCB2A+pCB1B::OM_5'_*_MP_* (lanes 1, 4, and 7), pOM2A+pOM1B (lanes 2, 5, and 8), or pOM2A+pOM1B::CB_3'_*_MP_* (lanes 3, 6, and 9) with *Bsr*GI or *Dde*I. The size of DNA after restriction enzyme digestion is indicated by base pair (bp). The *NSP-MP* fragment of ToLCNDV-CB after *Bsr*GI resulted in two bands of 1404 and 735 bp; digestion of the *NSP-MP* fragment of ToLCNDV-OM with *Bsr*GI resulted in a single 2101-bp band. The *Dde*I digestion of the *NSP-MP* fragments from ToLCNDV-OM or ToLCNDV-CB also resulted in different banding patterns.
