# Supplementary material for: A single amino acid substitution in the movement protein enables the mechanical transmission of a geminivirus
Source: Mol Plant Pathol. 2020 Feb 20;21(4):571–88. doi: 10.1111/mpp.12917 (PMC7060137; doi:10.1111/mpp.12917)
Supplement: Supplementary file 4 — TABLE S1 Characteristics of oligonucleotide primers used to amplify the viral DNA of the tomato leaf curl New Delhi virus (ToLCNDV)‐CB and ToLCNDV‐OM isolates in this study [file MPP-21-571-s004.docx]

**Table S1.** Characteristics of oligonucleotide primers used to amplify the viral DNA of the tomato leaf curl New Delhi virus (ToLCNDV)-CB and ToLCNDV-OM isolates in this study

| Primers | Sequence (5' to 3') | Tm (℃)^a^ | Target | Location^b^ | Size (bp) |
| --- | --- | --- | --- | --- | --- |
| FJJ2010-65 | CGGTAACAAATTAATAAATATCGACT | 65 | CB DNA-A | CB DNA-A nt 1036-1061 (F) | 1643 |
| FJJ2010-66 | CAATTCTGCCATTTAGTGTCCA | 65 | CB DNA-A | CB DNA-A nt 2657-2678 (R) |  |
| FJJ2010-67 | GTGGGATCCATTATTGCAC | 62 | OM DNA-A | OM DNA-A nt 122-140 (F) | 2046 |
| FJJ2010-92 | tacggggaataaagaagatgcg | 62 | OM DNA-A | OM DNA-A nt 2147-2168 (R) |  |
| FJJ2010-12 | CGACGAAACCATGTAATGTG | 59 | CB DNA-B | CB DNA-B nt 396-415 (R) | 1754 |
| FJJ2010-13 | CTACTGTCTTGCTAATTATGTCC | 59 | CB DNA-B | CB DNA-B nt 1355-1377 (F) |  |
| FJJ2010-59 | GTTTATGACATGAATGAAATTGTG | 50 | OM DNA-B | OM DNA-B nt 213-236 (F) | 2150 |
| FJJ2010-60 | TAGCGATTGGACAATACAC | 50 | OM DNA-B | OM DNA-B nt 2345-2363 (R) |  |
| FJJ2007-38 | CCCAGCGTGACTGGCAAAGC | 50 | OM DNA-A | OM DNA-A nt 1736-1755 (R) | 1463 |
| FJJ2007-44 | CCAGCAGATATCATCATTTC | 50 | OM DNA-A | OM DNA-A nt 292-311 (F) |  |
| FJJ2007-49 | GAGATATGCTTTAATTCA | 50 | OM *NSP* | OM DNA-B nt 312-329 (F) | 772 |
| FJJ2007-53 | GTTCTTGGGATGCAGTT | 50 | OM *NSP* | OM DNA-B nt 1068-1084 (R) |  |
| FJJ2011-57 | GCAATCGCAGTATCCATGGT | 67 | CB *NSP* | CB DNA-B nt 561-580 (F) | 595 |
| FJJ2011-58 | GAGAGTGTTTGAATATAGTCCTG | 67 | CB *NSP* | CB DNA-B nt 1134-1156 (R) |  |
| FJJ2011-59 | GCAATCGCAGTATCAATTCC | 66 | OM *NSP* | OM DNA-B nt 559-578 (F) | 595 |
| FJJ2011-60 | GAGAGTGTTTGAATATAGTCCAC | 66 | OM *NSP* | OM DNA-B nt 1132-1154 (R) |  |
| FJJ2011-61 | CATTGTTCTACTGTCTTGC | 62 | CB *MP* | CB DNA-B nt 1348-1366 (F) | 800 |
| FJJ2011-62 | GTCAATAGGAAATGATGGAG | 62 | CB *MP* | CB DNA-B nt 2129-2148 (R) |  |
| FJJ2011-63 | CATTGCTCTACAGTCTTCG | 66 | OM *MP* | OM DNA-B nt 1347-1365 (F) | 800 |
| FJJ2011-64 | GTCAACAGGAAATGAAGGGA | 66 | OM *MP* | OM DNA-B nt 2128-2147 (R) |  |
| FJJ2011-66 | GCAGTTTTCTAAATTAGTATTTCAC | 64 | IR of CB DNA-B | CB DNA-B nt 362-386 (R) | 504 |
| FJJ2011-70 | CTCTATGTAATTGGTGTCTGGA | 64 | IR of CB DNA-B | CB DNA-B nt 2576-2597 (F) |  |
| FJJ2011-68 | GCAGATTTATAATTTAGTATTTTAG | 62 | IR of OM DNA-B | OM DNA-B nt 360-384 (R) | 496 |
| FJJ2011-71 | TGTAATTGGCGTCTGGC | 62 | IR of OM DNA-B | OM DNA-B nt 2561-2577 (F) |  |

^a^ Annealing temperature.

^b^ F, forward primer; R, reverse primer.
