# Supplementary material for: A single amino acid substitution in the movement protein enables the mechanical transmission of a geminivirus
Source: Mol Plant Pathol. 2020 Feb 20;21(4):571–88. doi: 10.1111/mpp.12917 (PMC7060137; doi:10.1111/mpp.12917)
Supplement: Supplementary file 5 — TABLE S2 Characteristics of the oligonucleotide primers used to construct infectious clones of tomato leaf curl New Delhi virus [file MPP-21-571-s005.docx]

**Table S2.** Characteristics of the oligonucleotide primers used to construct infectious clones of tomato leaf curl New Delhi virus

| Primers^a^ | Sequence (5' to 3')^b^ | Location |
| --- | --- | --- |
| FJJ2007-13 | AAGCTCTAGAACGTCTCCGTCTTTGTCG | ToLCNDV DNA-A nt 2250-2273 |
| FJJ2007-15 | TGTGAATTCCGCTTGTTT | ToLCNDV DNA-B nt 1871-1888 |
| FJJ2009-23 | CTTGGTACCATTGGTCGCGCAGCGCAAA | OM DNA-B nt 2150-2168 |
| FJJ2009-25 | CTAGAGCTCTCCATGGCTCGAGTTCACTGAGATGGGAAAT | OM DNA-B nt 440-423 |
| FJJ2009-26 | GAACCATGGTGTTTCCGTTGCCACACATTTCCTATCCCAG | 5' end of severe *NSP* gene |
| FJJ2009-27 | CATTTCCTATCCCAGCCAAATGGCTCTTTCTTCT | 5' end of OM *NSP* gene with the extract sequences of 5' end of severe *NSP* gene |
| FJJ2009-32 | CGTGCTAGCGTCATCCAATGTAATTAAG | OM DNA-B nt 1230-1247 |
| FJJ2009-33 | CCAGCTAGCTGTATTGAAATGCACTAG | OM DNA-B nt 1248-1265 |
| FJJ2009-34 | CGTTGAGCTCTGGATAAAATTCAAACAA | OM DNA-B nt 338-355 |
| FJJ2010-95 | CGAGCTCCAGAAACACAGAAGGACGGAC | IR of CB and OM DNA-B |
| FJJ2010-96 | CGGGATCCGTAATGTGATATATGTAGCAG | IR of CB DNA-B |
| FJJ2010-97 | CGGGATCCTCACTGAGATGGGAAATGTGTG | IR of OM DNA-B |
| FJJ2010-98 | CGGGATCCATGGTTTCGTCGTTGCCACAC | 5' end of CB *NSP* gene |
| FJJ2010-99 | CGGGATCCAATGGCTCTTTCTTCTCC | 5' end of OM *NSP* gene |
| FJJ2010-100 | GTAGATATCCATCATCCAATGTAATTAAG | 3' end of CB and OM *NSP* genes |
| FJJ2010-101 | CAGGATATCTATTGAAATGCACTAGAAAC | 5' end of CB and OM *MP* genes |
| FJJ2010-102 | CCCAAGCTTACTCCTGCTTCTCTCTAAC | 3' end of CB and OM *MP* genes |
| FJJ2010-103 | CCCAAGCTTTTCTCTCTCTAGACACC | IR of CB and OM DNA-B |
| FJJ2010-104 | ACGCGTCGACGTTTGAATTAAAGCATATCTC | IR of CB and OM DNA-B |

^a^ The annealing temperature for all primers was around 50 ℃.

^b^ Restriction enzyme sites (underlined) were incorporated into most of the primers to facilitate cloning. GGTACC: *Kpn*I; GAGCTC: *Sac*I; CCATGG: *Nco*I; CTCGAG: *Xho*I; GCTAGC: *Nhe*I; GGATCC: *Bam*HI; GATATC: *Eco*RV; AAGCTT: *Hin*dIII; GTCGAC: *Sal*I. The 39-nt sequence from ToLCNDV-severe isolate is indicated by the gray box.
